# Mass spectrometry imaging-based metabolomics to visualize the spatially resolved reprogramming of carnitine metabolism in breast cancer

**DOI:** 10.7150/thno.45543

**Published:** 2020-05-30

**Authors:** Chenglong Sun, Fukai Wang, Yang Zhang, Jinqian Yu, Xiao Wang

**Affiliations:** 1 School of Pharmaceutical Sciences, Qilu University of Technology (Shandong Academy of Sciences), Jinan 250014, China.; 2 Shandong Analysis and Test Center, Qilu University of Technology (Shandong Academy of Sciences), Jinan 250014, China.; 3 Shandong Cancer Hospital and Institute, Shandong First Medical University and Shandong Academy of Medical Sciences, Jinan 250117, China.; 4 Department of Ultrasound in Medicine, Shanghai Jiao Tong University Affiliated Sixth People's Hospital, Shanghai 200233, China.

**Keywords:** mass spectrometry imaging, metabolomics, carnitines, metabolic reprogramming, breast cancer

## Abstract

New insights into tumor-associated metabolic reprogramming have provided novel vulnerabilities that can be targeted for cancer therapy. Here, we propose a mass spectrometry imaging (MSI)-based metabolomic strategy to visualize the spatially resolved reprogramming of carnitine metabolism in heterogeneous breast cancer.

**Methods:** A wide carnitine coverage MSI method was developed to investigate the spatial alternations of carnitines in cancer tissues of xenograft mouse models and human samples. Spatial expression of key metabolic enzymes that are closely associated with the altered carnitines was examined in adjacent cancer tissue sections.

**Results:** A total of 17 carnitines, including L-carnitine, 6 short-chain acylcarnitines, 3 middle-chain acylcarnitines, and 7 long-chain acylcarnitines were imaged. L-carnitine and short-chain acylcarnitines are significantly reprogrammed in breast cancer. A classification model based on the carnitine profiles of 170 cancer samples and 128 normal samples enables an accurate identification of breast cancer. CPT 1A, CPT 2, and CRAT, which are extensively involved in carnitine system-mediated fatty acid β-oxidation pathway were also found to be abnormally expressed in breast cancer. Remarkably, the expressions of CPT 2 and CRAT were found for the first time to be altered in breast cancer.

**Conclusion:** These data not only expand our understanding of the complex tumor metabolic reprogramming, but also provide the first evidence that carnitine metabolism is reprogrammed at both the metabolite and enzyme levels in breast cancer.

## Introduction

Breast cancer is the most commonly diagnosed cancer and the leading cause of cancer incidence and mortality in women worldwide, with more than 2.0 million new cases and 0.6 million deaths each year 1. There is growing evidence that metabolic reprogramming is an essential hallmark for cancer cells to grow and proliferate under hostile conditions; this is likely to contribute to resistance against therapeutics 2,3. A better understanding of the complex metabolic reprogramming in specific cancer tissue is key to defining pathways that are most limiting for cancer progression 4-7. The carnitine system including L-carnitine, acylcarnitines, and related enzymes is an important mediator in cancer metabolic networks. It intertwines crucial metabolites, pathways, and factors that supply biosynthetic and energetic demands for cancer cells 8,9. L-carnitine serves an indispensable role in fatty acid transport into the mitochondrial matrix for β-oxidation and maintains the homeostasis of coenzyme A (CoA) in mitochondrial pool 10,11. CoA availability is crucial for the biofunctions of multiple metabolic pathways such as tricarboxylic acid cycle, amino acid metabolism, and pyruvate oxidation 12,13. Acylcarnitines are closely related to the balance regulation of intracellular sugar and lipid metabolism 14-16.

Liquid chromatography-mass spectrometry (LC-MS) has made significant inroads into profiling the metabolic signatures of L-carnitine and acylcarnitines in cancer samples 17,18. Unfortunately, the spatial distribution discrepancy of carnitines in heterogeneous cancer tissue is frequently lost in LC-MS-based studies in which the analysis is performed on a tissue homogenate. The metabolism and transport of endogenous metabolites in cancer tissues are all dynamic and spatially resolved; therefore, knowing the spatial distributions of carnitines at the complex tumor microenvironment is imperative. Matrix-assisted laser desorption/ionization-mass spectrometry imaging (MALDI-MSI) is a label-free technique to characterize the spatial distributions of multiple endogenous species in tissues 19-26. Since it was first introduced, MALDI-MSI has been increasingly adopted for profiling the spatiotemporal signatures of cancer-associated metabolic changes 27-33. Previous studies have demonstrated that imaging the spatial distributions of carnitines in cancer tissues was feasible, but the number of carnitines imaged was limited to L-carnitine and a few high-abundance acylcarnitines 34,35. Notably, carnitines represent a large class of metabolites interconnected in metabolic pathways, and the levels of different carnitines vary greatly. Thus, a comprehensive profiling of carnitines in heterogeneous cancer tissue is crucial for our understanding of cancer metabolism which may provide fundamental insights into deciphering the possible role of the carnitine system in cancer development and progression.

**Scheme 1:**
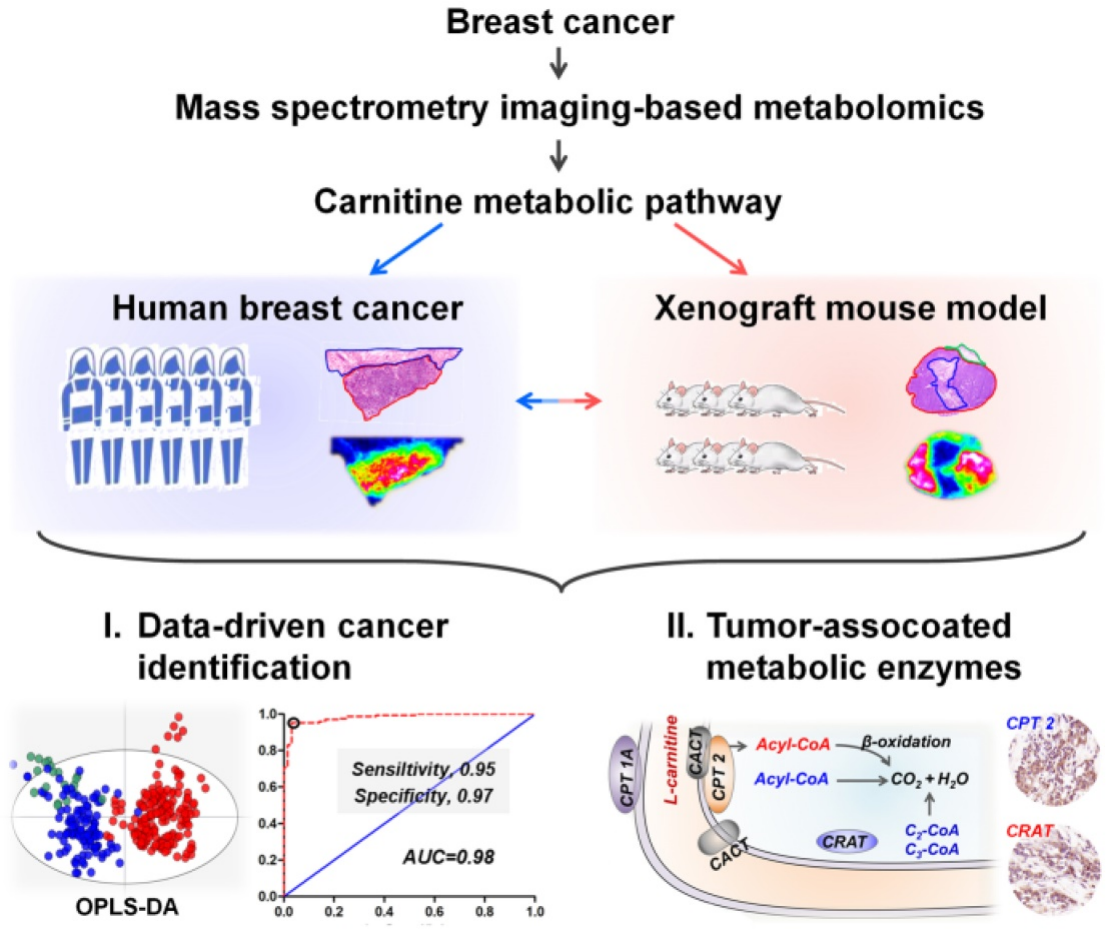
Schematic illustrations showing the mass spectrometry imaging based metabolomic strategy to visualize the reprogramming of carnitine metabolism in breast cancer.

Here, we propose a mass spectrometry imaging-based metabolomic strategy to visualize the spatially resolved reprogramming of carnitine metabolism in breast cancer. Metabolomic investigations performed at both human and xenograft mouse model levels all indicate that L-carnitine and short-chain acylcarnitines are significantly reprogrammed in breast cancer tissues. Moreover, carnitine system-mediated fatty acid β-oxidation pathway was also found to be altered in breast cancer tissues. Three abnormally expressed metabolic enzymes including carnitine palmitoyltransferase 1A (CPT 1A), carnitine palmitoyltransferase 2 (CPT 2), and carnitine acetyltransferase (CRAT), which are directly involved in fatty acid β-oxidation were further discovered. The spatially resolved profiling of the altered carnitine metabolism in breast cancer tissues, from metabolites to enzymes, expands our understanding of tumor metabolic reprogramming. The design of this study is shown in Scheme 1.

## Materials and Methods

### Reagents and antibodies

Tween-20 and Triton X-100 were obtained from Beijing Solarbio Science & Technology Co., Ltd. (Beijing, China). Bovine Serum Albumin was purchased from Sigma (St. Louis, USA). PV-9000 two-step immunohistochemical kit and DAB kit were provided by Beijing Zhongshan Goldenbridge Biotechnology Company (Beijing, China). Anti-CPT1A antibody (ab220789) and Anti-CPT2 antibody (ab181114) were obtained from abcam. Anti-CRAT (HPA019230) and anti-CROT (HPA019364) antibodies were purchased from Atlas Antibodies. Ultrapure water was obtained from a Milli-Q Water System (Millipore Corporation, Bedford, MA, USA).

### Animal studies

The animal experiments were approved by the Ethics Committee of Shandong Analysis and Test Center (Jinan, China). For xenograft experiments, female Balb/c-nu/nu mice (18-20 g) were provided by Vital River Lab Animal Technology Co., Ltd. (Beijing, China). MDA-MB-231 cells were cultured in L-15 medium and supplemented with 10% fetal bovine serum. Tumor implantation was carried out by injecting 100 microliters of the final cell suspension (1×10^7^ cells) into the right back of mice. The experiment was ended when the tumor volume reached approximately 300 mm^3^. Tumor and the surrounding normal tissues were acquired after sacrifice via anesthesia. Samples were then flash-frozen in liquid nitrogen for 20 s and stored at -80 °C.

### Human breast cancer tissue sample collection

A total of 58 pairs of human breast cancer tissue samples including cancer tissue and adjacent normal tissue were collected by surgical resection, having been previously approved by the local Ethical Review Board of Shandong Cancer Hospital (Jinan, China); all cohort patients provided written informed consent. The detailed demographic and clinical characteristics are demonstrated in Table S1. All samples were collected from the middle of the tumor. The diameter of these samples is generally 1-2 cm. The collected breast cancer tissues were first flash-frozen in liquid nitrogen for 20 s and then transferred to cryogenic vials and stored at -80 °C.

### Tissue sample processing

All of the tissue samples were cut into 10 μm frozen sections at -25 °C on a cryostat microtome (Thermo CryoStar NX50 NOVPD, Bremen, Germany). To obtain more comprehensive tissue information, each human breast cancer tissue sample was sectioned from three different locations. Thus, 170 cancer samples and 128 normal samples were obtained from 58 human breast cancer patients. Two sets of tissue sections were thaw-mounted onto indium tin oxide (ITO)-coated glass slides for MALDI-MSI analysis. One set of tissue section was fixed in 4% paraformaldehyde and stained by hematoxylin-eosin (H&E). Four sets of tissue sections were thaw-mounted onto normal microscope slide for immunohistochemical analysis. To image more carnitines with stronger ion intensities, ten adjacent tissue sections from one cancer tissue were prepared to optimize the washing solvents. One of the samples had no preprocessing, and the other four were immersed in 50% ethanol, 70% ethanol, 90% ethanol, and 100% ethanol. The results suggest that 100% ethanol washing greatly enhances the sensitivities of short-chain acylcarnitines. Five adjacent tissue sections were then used to optimize washing time.

### Matrix coating

Matrix coating used an HTX TM-Sprayer^TM^ (HTX Technologies, Carrboro, NC). 1,5-diaminonaphthalene, 2.5 mg/mL in acetonitrile-H_2_O (70:30, *v*/*v*), was optimized as MALDI matrix for carnitine imaging. A total of eight spray cycles were made over the tissue section with a flow rate of 0.075 mL/min at 55 °C. The track speed and track spacing were set to 800 mm/min and 3 mm, respectively.

### MALDI-MSI analysis

After drying in vacuum for 15 min, the cryosections were subjected to MALDI-MSI analysis by using a Rapiflex MALDI tissuetyper^TM^ TOF/TOF MS (Bruker Daltonics, Billerica, MA) equipped with a smartbeam^TM^ 3D laser. The laser was fired at a repetition rate of 5000 Hz. The mass spectral data were acquired in both positive and negative ion modes over the *m/z* range of 80-1000, and the spatial resolution was set to 100 μm. The MS images were viewed by using FlexImaging 5.0 software (Bruker Daltonics) and SCiLS Lab 2018b software (GmbH, Bremen, Germany).

### Data analysis

Raw MALDI-MS spectra were imported into SCiLS Lab 2018b software to construct MS image and perform segmentation analysis. The region-specific MS profiles were precisely extracted by matching ion images with H&E stain images. Two-dimensional dataset matrixes were built by using Markerview^TM^ software 1.2.1 (AB SCIEX, USA) with mass tolerance *m/z* 0.01. SIMCA-P 14.0 software package (Umetrics AB, Umeå, Sweden) was used for multivariate statistical data analysis, including partial least squares discrimination analysis (PLS-DA) and orthogonal PLS-DA (OPLS-DA). Receiver Operating Characteristic (ROC) curve, logistic regression, and the Student's t-test analysis were performed on SPSS 21.0 and GraphPad Prism 6.0. Data-driven segmentation analysis, pixel-to-pixel correlation analysis, and *in situ* principal component analysis (PCA) were performed via SCiLS Lab software.

### Immunohistochemistry

Expression of CPT 1A, CPT 2, CRAT, and CROT in the human breast cancer tissue sections which adjacent to the ones analyzed by MALDI-MSI were assessed. The frozen tissue sections were first fixed in 4% paraformaldehyde for 10 min. Then, the sections were immersed in 0.25% Triton X-100 for 15 min and blocked with 1% bovine serum albumin for 30 min. After incubated with targeted antibodies (1:200 for CPT 1A, 1:50 for CPT 2, 1:100 for CRAT, and 1:50 for CROT) at 4 °C overnight, the spatial expressions of these four metabolic enzymes in breast cancer tissue sections were characterized using a PV-9000 two-step IHC kit and DAB kit. Images were taken with a Pannoramic MIDI scanner (3DHISTECH, Budapest, Hungary) and analyzed by Image-Pro Plus software (IPP, version 6.0, Silver Spring, MD, USA).

### Analyte identification

The adducted ions of carnitines and other metabolites were first compared with free databases Metlin (http://metlin.scripps.edu) and Human Metabolome Database (http://hmdb.ca/) using exact molecular weights with a mass error of less than 5 ppm. High-resolution tandem MS experiments were then performed on an orbitrap mass spectrometer (Q Exactive, Thermo Scientific, Bremen, Germany). Analyte identification was further carried out based on isotope distributions and MS/MS spectra. The detailed operation process, MS/MS data and the structure-specific pattern ions of the target metabolites are listed in Supplementary Material (Figures S14-S23).

## Results and Discussion

### MALDI-MSI-driven breast cancer heterogeneous characterization

Human breast cancer tissue section can be divided into cancer tissues (CT) and paracancerous normal tissues (NT). We first performed untargeted MALDI-MSI imaging in positive ion mode over the *m/z* range of 80-1000. CT- and NT-specific mass spectra were precisely extracted based on the overlay image of optical and MS images (Figure S1). These data suggest that the mass profiles of CT and NT are quite different, representing that the underlying metabolites of breast cancer cells have undergone tremendous changes compared to normal cells.

**Figure 1:**
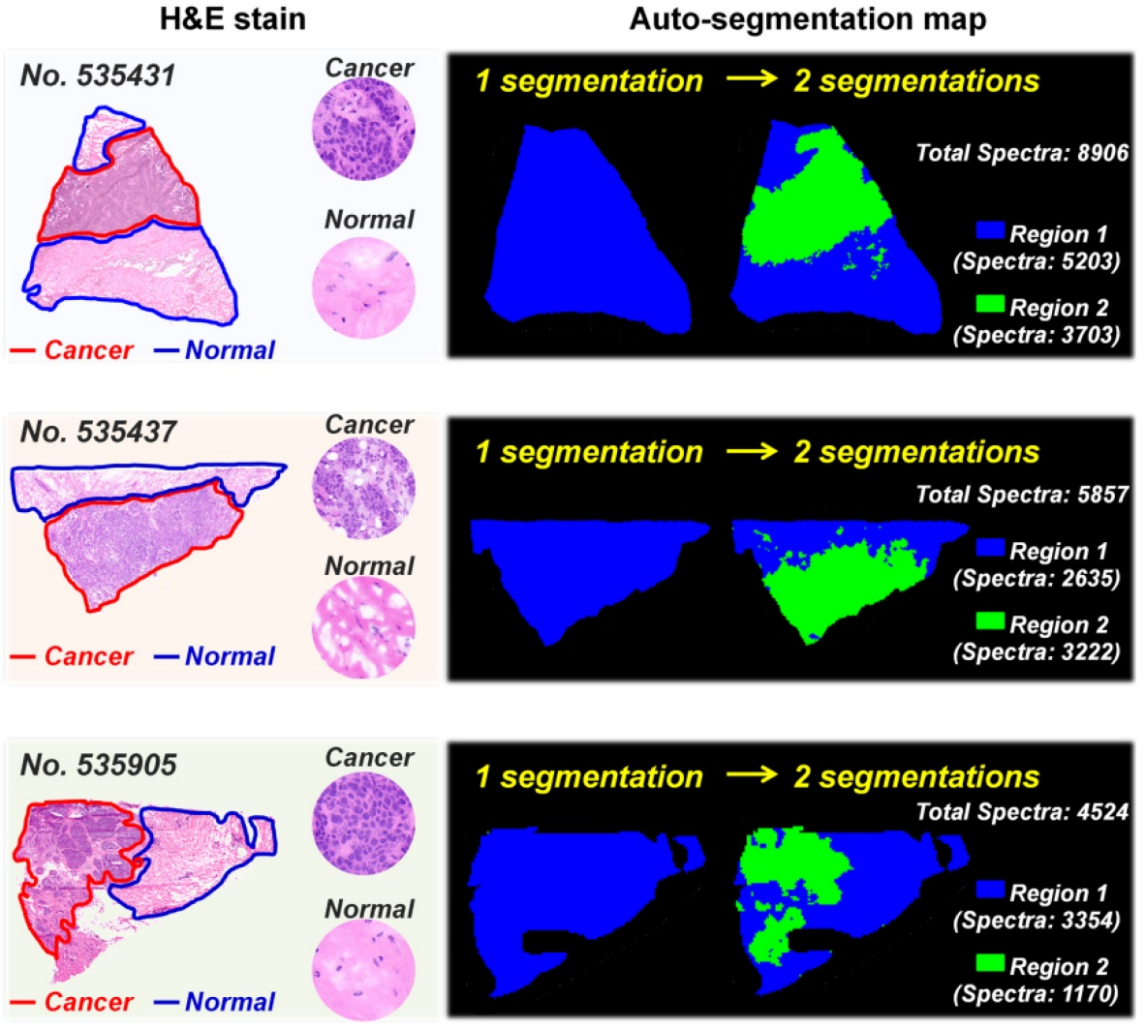
Auto-segmentation of breast cancer tissue sections (These three tissue sections are from patient *No*. 535431, *No*. 535437, and *No*. 535905. The pixels colored in green have similar spatial features with cancerous regions).

MS imaging is an effective way to study cancer metabolic heterogeneity by directly mapping the spatial distributions of metabolites. In fact, each pixel in tissue MS images has its region-specific metabolic fingerprints, and these metabolic fingerprints can reflect the structural and functional characteristics of tissue 36. Here, we determined the metabolic similarities of different pixels in breast cancer tissue MS images via the segmentation function in SCiLS Lab software. Image pixels with similar metabolic fingerprints were classified as one group via bisecting k-means clustering; each group was then assigned selected colors and displayed as label maps. Figure 1 demonstrates the reconstructed label maps of three breast cancer tissue sections. The results suggest that the image pixels can be divided into two clusters based on their metabolic fingerprints. Significantly, the reconstructed label maps can clearly characterize the cancerous regions and normal regions of breast cancer tissue sections. The pixels colored in green have similar spatial features with cancerous regions, while the spatial distributions of blue pixels are consistent with normal regions.

To further explore the metabolic difference between breast CT and NT, we performed unsupervised principal component analysis (PCA) based on the *in situ* MS spectra. As shown in Figure S2, the calculated score plots exhibited a clear separation between CT and NT. The levels of L-carnitine and a variety of short-chain acylcarnitines such as acetylcarnitine, acylcarnitine C3:0, and acylcarnitine C4:0 in CT were significantly higher than that in NT (Figure S3A). Moreover, correlation analysis suggests that L-carnitine, acetylcarnitine, acylcarnitine C3:0, and acylcarnitine C4:0 in breast cancer tissue showed very strong positive correlation, with correlation coefficients ranging from 0.803 to 0.953 (Figure S3B). As an indispensable endogenous metabolite, carnitines not only enhance cell antioxidant activity, but also participate in cell energy metabolism 37. The elevated energy metabolism is an important hallmark of cancer 2. Previous studies have also shown that carnitine can be used as a potential generic prognostic biomarker in some tumors such as sarcoma 38. Therefore, we speculate that the metabolic reprogramming of carnitines may be related to the development of breast cancer. Although some carnitines can be detected, it is still difficult to effectively detect those low-abundance ones such as acylcarnitine C6:0 and acylcarnitine C7:0 (Figure S4). Improving the sensitivity of MALDI-MSI to image more carnitines will provide significant clues for understanding the reprogramming of carnitines in breast cancer.

### 100% ethanol washing enhances the MALDI-MS imaging of carnitines

Finding the appropriate organic solvent to wash away unwanted components in tissue sections has proven to be an effective way to improve the MS imaging signals of targeted molecules 39-41. Gradient ethanol (70% ethanol followed by 95% ethanol) is the most commonly used washing strategy and can remove as many phospholipids as possible from biological tissues. However, our data indicate that gradient ethanol washing also results in the loss of carnitines and other low-molecular-weight metabolites (*m/z* < 500, Figure S5). We compared the performance of 50% ethanol, 70% ethanol, 90% ethanol, and 100% ethanol washing on the MS imaging of carnitines in breast cancer tissues, and the results suggest that 50% ethanol, 70% ethanol, and 90% ethanol washing significantly reduce the ion signals of carnitines, 100% ethanol washing greatly improves the signal intensities of carnitines (Figure S6). The impact of washing time on MS imaging of carnitines was also studied. Figure S7 shows that all carnitines demonstrated the strongest ion intensities when the tissue section was washed in 100% ethanol for 60 s or 90 s. Further increases in washing time not only reduce the carnitines' ion signals but also lead to significant molecular delocalization. This may be attributed to the *in situ* dissolution of carnitines from tissue sections.

**Figure 2:**
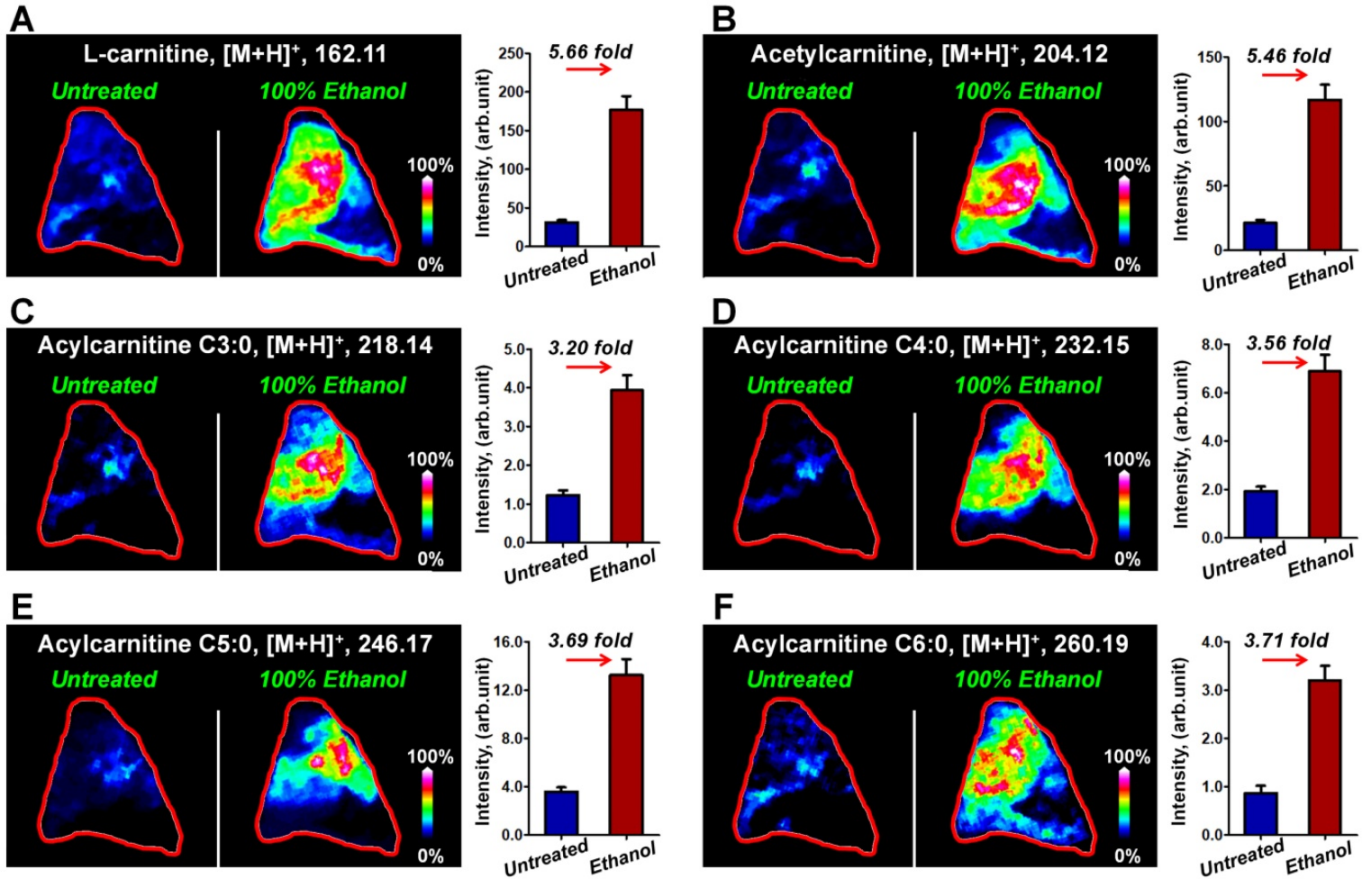
MS images of L-carnitine **(A)** and five acylcarnitines **(B-F)** in untreated and 100% ethanol-washed breast cancer tissue section (These tissue sections are from patient *No*. 535431).

Figure 2 illustrates the typical MS images of L-carnitine and five acylcarnitines in untreated breast cancer tissue section and tissue sections that have been washed in 100% ethanol for 90 s. The results suggest that the ion signals of L-carnitine and acetylcarnitine in 100% ethanol-washed section increased by 5.66 times and 5.46 times (Figure 2A and 2B), respectively. The ion signals of acylcarnitine C3:0, acylcarnitine C4:0, acylcarnitine C5:0 and acylcarnitine C6:0 all increased by more than three-fold (Figure 2C-2F). Remarkable, 100% ethanol washing enables the MS imaging of some acylcarnitines such as acylcarnitine C7:0 and acylcarnitine C8:0 in breast cancer tissue. This cannot be achieved without washing (Figure S8). Using this organic washing protocol, a total of 17 carnitines, including L-carnitine, 6 short-chain acylcarnitines (C*_n_*, 2-5), 3 middle-chain acylcarnitines (C*_n_*, 6-10), and 7 long-chain acylcarnitines (C*_n_*, 12-18) can be detected and imaged in an untargeted run (Figure S9). To the best of our knowledge, this is the largest number of carnitines yet detected by mass spectrometry imaging.

### MALDI-MS imaging highlights the abnormally expressed carnitines in the breast cancer xenograft mouse model

**Figure 3:**
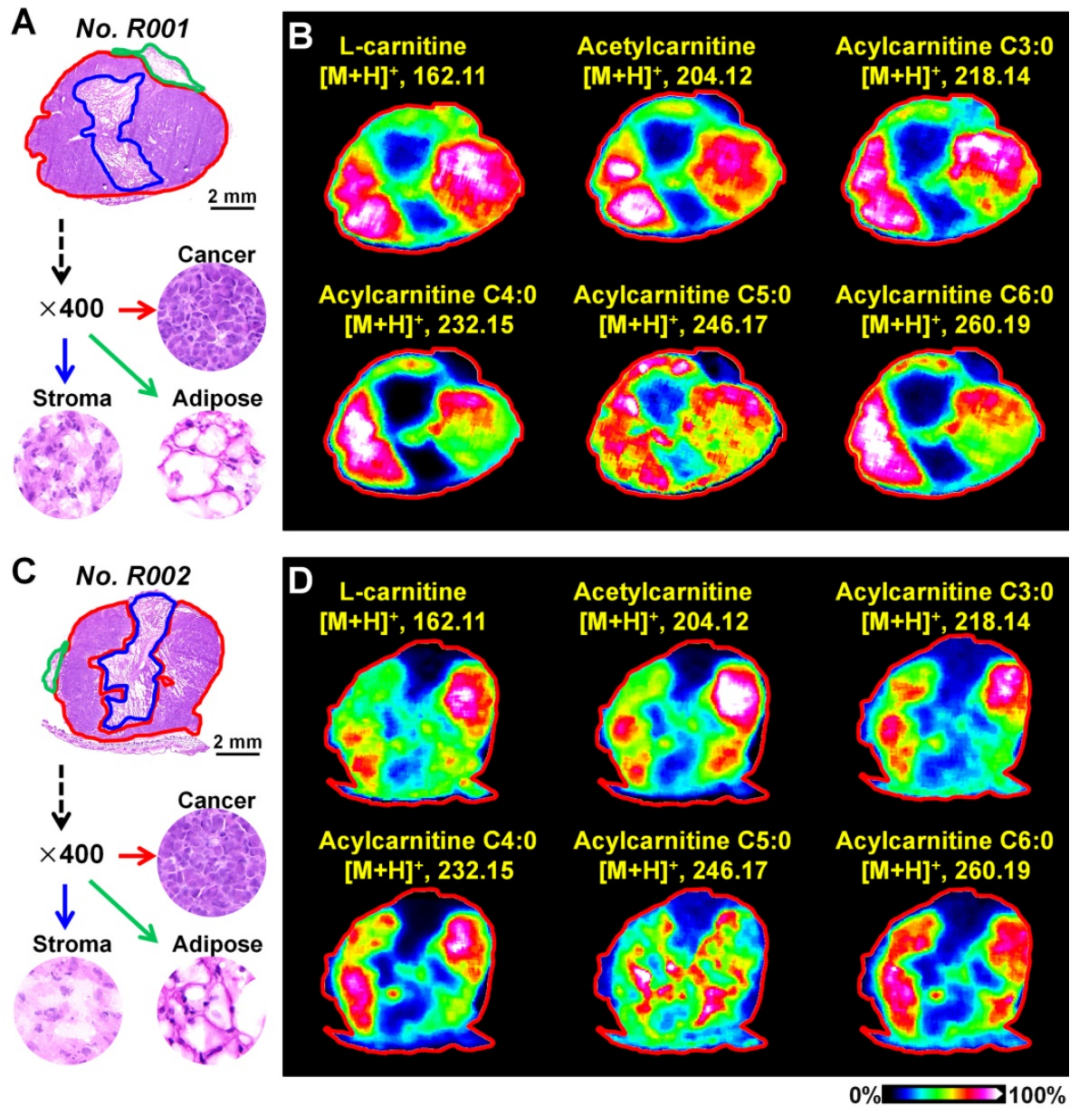
**A)** The H&E stain of mouse breast cancer tissues (*No*. R001); **B)** MS images of L-carnitine and five acylcarnitines in mouse breast cancer tissues (*No*. R001); **C)** H&E stain of mouse breast cancer tissues (*No*. R002);** D)** MS images of L-carnitine and five acylcarnitines in mouse breast cancer tissues (*No*. R002).

Breast cancer tissues from xenograft tumor mouse models were analyzed using the optimized MALDI-MS imaging method. As shown in Figure 3A and 3C, heterogeneous cancer tissue can be further divided into the cancer region, stroma region, and adipose region according to cell components. Region-specific mass spectra were extracted based on the overlay of optical and MS images. A PCA score plot built from the region-specific mass spectra presents a clear separation between cancer and other regions (Figure S10). We extracted the MS images of L-carnitine and acetylcarnitine in mouse breast cancer tissues suggesting that the levels of L-carnitine, acetylcarnitine, acylcarnitine C3:0, acylcarnitine C4:0, acylcarnitine C5:0 and acylcarnitine C6:0 in the cancerous region were significantly higher than those in adjacent stroma and adipose regions (Figure 3B and 3D). These data show that the alteration of carnitine is an important component of breast cancer metabolic reprogramming.

### MALDI-MS imaging of the altered carnitine metabolism in human breast cancer

**Figure 4:**
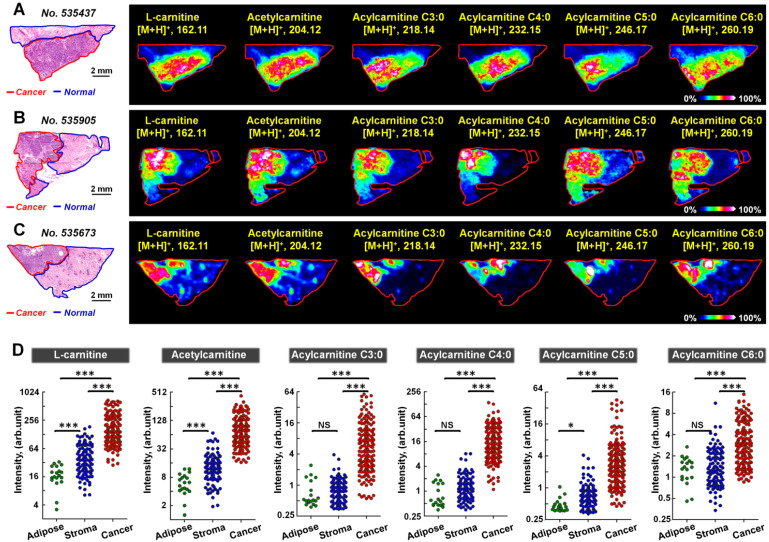
** A-C)** MS images of six carnitines in human breast cancer tissues (these three tissue sections are from patient *No*. 535437, *No*. 535905, and *No*. 535673); **D)** Statistical data of six carnitines in 170 human breast cancer tissues and 128 paired normal tissues (including 109 normal stromal tissues and 19 normal adipose tissues); the scale of the *y*-axis is log 2; ***, *p* < 0.001; **, *p* < 0.01; *, *p* < 0.05; NS, no significant differences.

We further performed MALDI-MS imaging on postoperative human breast cancer specimens containing both cancer tissues and adjacent normal tissues. A total of 170 cancer samples and 128 normal samples were extracted from 58 breast cancer patients. The 128 normal samples can be further divided in 109 normal stromal tissues and 19 normal adipose tissues. Figure 4 demonstrates the MS images of L-carnitine, acetylcarnitine, acylcarnitine C3:0, acylcarnitine C4:0, acylcarnitine C5:0, and acylcarnitine C6:0 in three human breast cancer tissue sections. The results suggest that the expressions of all these six carnitines in cancer regions were significantly higher than that in the paired normal regions. The statistical results of these six carnitines based on 170 cancer samples and 128 normal samples are shown in Figure 4D. In comparison to normal tissues, the levels of L-carnitine and acetylcarnitine in cancer tissue increased by 4.46 and 6.36 times, respectively. Acylcarnitine C3:0 and acylcarnitine C4:0 were both significantly upregulated by more than 10-fold in cancer tissues: acylcarnitine C3:0 by a factor of 10.01 and acylcarnitine C4:0 by 14.26. Acylcarnitine C5:0 and acylcarnitine C6:0 increased by 4.95 and 2.26 times in cancer tissues, respectively. The statistical results of other acylcarnitines were illustrated in Figure S11. Although the content of acylcarnitine C7:0 was found to be higher in cancer tissues, there was no significant difference in the contents of other acylcarnitines including acylcarnitine C8:0, acylcarnitine C16:0, acylcarnitine C16:1, acylcarnitine C16:2, acylcarnitine C18:0, acylcarnitine C18:1, and acylcarnitine C16:2 in cancer tissues and adjacent normal tissues. These data suggest that L-carnitine and short-chain acylcarnitines are the main types of altered carnitines in human breast cancer.

**Figure 5:**
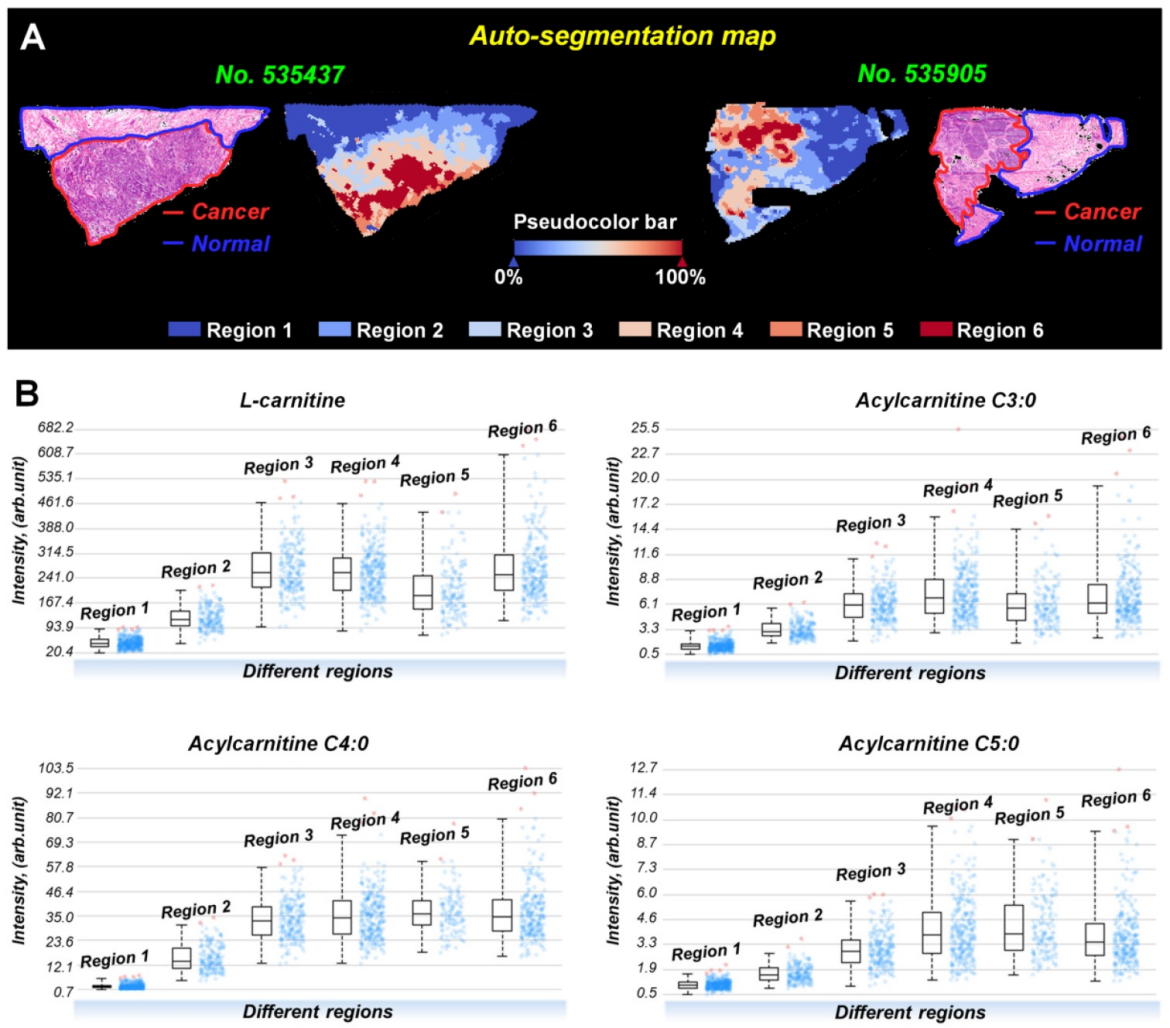
**A)** Data-driven segmentation analysis of breast cancer tissues (These two tissue sections are from patient *No*. 535437 and *No*. 535905); **B)** Ion intensities of L-carnitine, acylcarnitine C3:0, acylcarnitine C4:0, and acylcarnitine C5:0 in different segmentation-derived regions (The statistical data comes from all pixels of sample *No.* 535437).

Interestingly, the spatial distributions and contents of carnitines are not completely consistent even within the same tissue region (Figure S12). To investigate this metabolic heterogeneity in breast cancer tissue, we performed data-driven segmentation analysis based on the underlying metabolic fingerprints of each image pixel. The segmentation results indicate that the breast cancer tissue sections can be divided into six different regions (Figure 5A). These six regions are colored according to their global metabolite profiles. As the global metabolite profiles become closer, their colors match on the pseudocolor bar and vice versa (see arrows in Figure 5A). Significantly, our data suggest that the assigned colors of different segmentation-derived regions are closely related to their distance to the cancer center (region 6). The color of the regions near the cancer center is similar to that of the cancer center. The color difference between them and cancer center becomes larger as the spatial distance increases. This means that the metabolite profile of breast cancer tissue was changed in a stepwise way from the cancer center (region 6) to distal normal (region 1).

PCA analysis based on the metabolite profiles of these six segmentation-derived regions was also performed. Figure S13 shows that the score plots had a progressive trend from region 1 to region 6, indicating a stepwise metabolic change from cancer center to distal normal. The carnitine profiles of these six segmentation-derived regions were also extracted. Figure 5B shows the levels of L-carnitine, acylcarnitine C3:0, acylcarnitine C4:0, and acylcarnitine C5:0 in different regions. In general, the contents of these carnitines gradually change over segmentation-derived regions with the highest content in cancer center region (region 6) and the lowest in the distal normal region (region 1). These data suggest that the cancer center may be the region with the most severe metabolic reprogramming of carnitines in all of the breast cancer tissue. A Previous study indicated that the central region of the tumor always experiences increased hypoxic and oxidative stress 42. The alterations of carnitines in cancer center regions probably occurred because of the inhibition of β-oxidation during hypoxia, leading to the accumulation of carnitines in the cytoplasm of hypoxic tumor cells.

### Distinguishing breast cancer tissue from adjacent normal tissue based on the carnitines

To explore the discriminating ability of carnitines in distinguishing cancerous tissue from normal tissue, we built an OPLS-DA model based on the carnitine profiles of 170 cancer samples, 109 normal stromal tissues, and 19 normal adipose tissues. Notably, the detected ion intensity of different carnitines in this study varies by more than 300 times. Previous studies showed that the ions with stronger intensities have a greater influence on multivariate statistical analysis, resulting in the neglect of low-content ones 43,44. Therefore, we performed log transformation before OPLS-DA analysis to alleviate the dependence of heteroscedasticity on the ion signal intensity.

**Figure 6:**
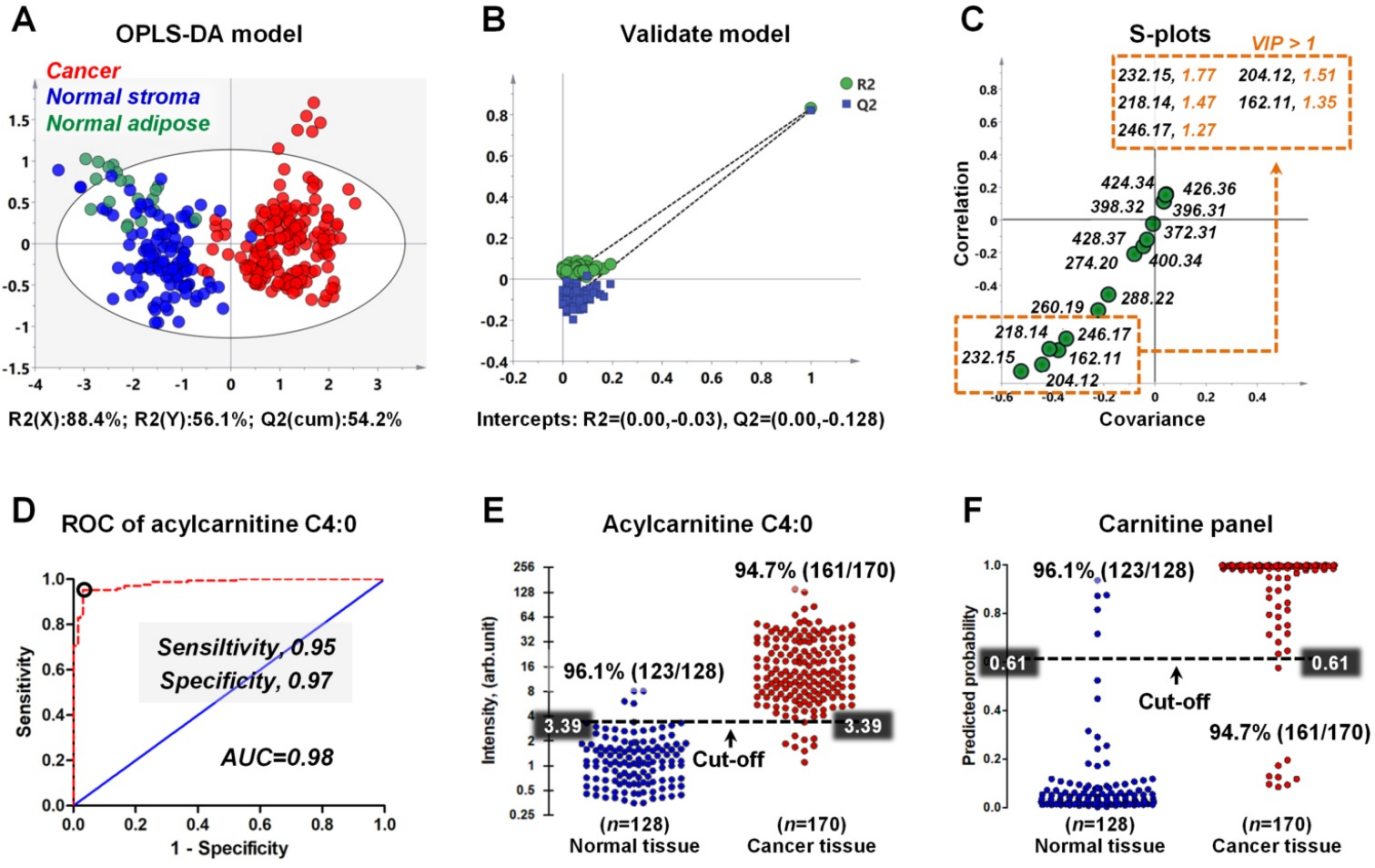
**A)** OPLS-DA score plots for 170 human breast cancer tissues and 128 paired normal tissues (including 109 normal stromal tissues and 19 normal adipose tissues); **B)** The validation plot obtained from 100 permutation tests; **C)** The S-plots of the OPLS-DA model; **D)** ROC curve for distinguishing cancer tissue from normal tissue based on the ion intensity of acylcarnitine C4:0; **E)** Discriminating efficiency of breast cancer based on the ion intensity of acylcarnitine C4:0; **F)** Discriminating efficiency of breast cancer based on the ion intensity of carnitine panel.

As shown in Figure 6A, although there is no obvious clustering between normal stroma and normal adipose, a clear indication of separation was observable between cancer and normal tissues (including normal stroma and normal adipose). This result suggests that the reprogrammed carnitines can distinguish cancer tissues from normal tissue. To assess validity of the OPLS-DA model, we performed a random permutation test with PLS-DA model, corresponding to the OPLS-DA model across same components. The PLS-DA model was validated by an iterative seven-round cross-validation with 1/7th of the samples being excluded from the mode in each round. Validation with 100 random permutation tests generated intercepts of Q2 = -0.128 and R2 = -0.03 (Figure 6B). This data strongly support the validity of the OPLS-DA model, since the intercept for Q2 to the *y*-axis was less than 0 (blue box), and all permuted R2 values (green circle in the left) are lower than the original point of the R2 value (green circle in the right). An S-plot showing the covariance and correlation between the carnitines and the modeled class designation was used to identify statistically significant variables. Selection of variables with a high covariance and high correlation values was preferred 45. Here, acylcarnitine C4:0 (*m/z* 232.15) has the largest covariance and correlation values, so it was identified as the most significant variable to distinguish cancer tissues from normal tissues (Figure 6C). Other carnitines such as acetylcarnitine (*m/z* 204.12), acylcarnitine C3:0 (*m/z* 218.14), L-carnitine (*m/z* 162.12), and acylcarnitine C5:0 (*m/z* 246.17) also exhibited high covariance and correlation values. The variable importance in the projection (VIP) value, a parameter used to reflect the influence of variable on the classification, was shown in Figure 6C. Variables with a VIP > 1 had an above average influence on the explanation of the classification. This also indicates that acylcarnitine C4:0 is the most important carnitine to distinguish cancer and normal tissues.

Receiver operating characteristic curve (ROC) was constructed to evaluate the discrimination performance of acylcarnitine C4:0. Figure 6D indicates that the ROC curve generated from acylcarnitine C4:0 exhibited good discrimination ability with an area under curve (AUC) of 0.98, with sensitivity and specificity values of 0.95 and 0.98. On the basis of the highest prediction sensitivity and specificity of the ROC, the optimal cut-off values were 3.39 arbitrary units for cancer tissues versus normal tissues (*n* = 298) (Figure 6E). At this cut-off value, 123 of 128 normal breast tissue samples were correctly classified, and 161 of 170 breast cancer tissue samples were correctly classified (Figure 6E). Furthermore, we combined the five carnitines with VIP > 1 into one carnitine panel by logistic regression, and then performed ROC analysis on this carnitine panel (the five carnitines are shown in the illustration in Figure 6C). The prediction probability value of carnitine panel is shown in Figure 6F. The accuracy of the carnitine panel was the same as that of acylcarnitine C4:0 at the optimal cut-off value and both had an accuracy of 96.1% for normal breast tissues and 94.7% for breast cancer tissues.

### *In situ* discovery and validation of altered metabolic enzymes in carnitine metabolic pathway

**Figure 7:**
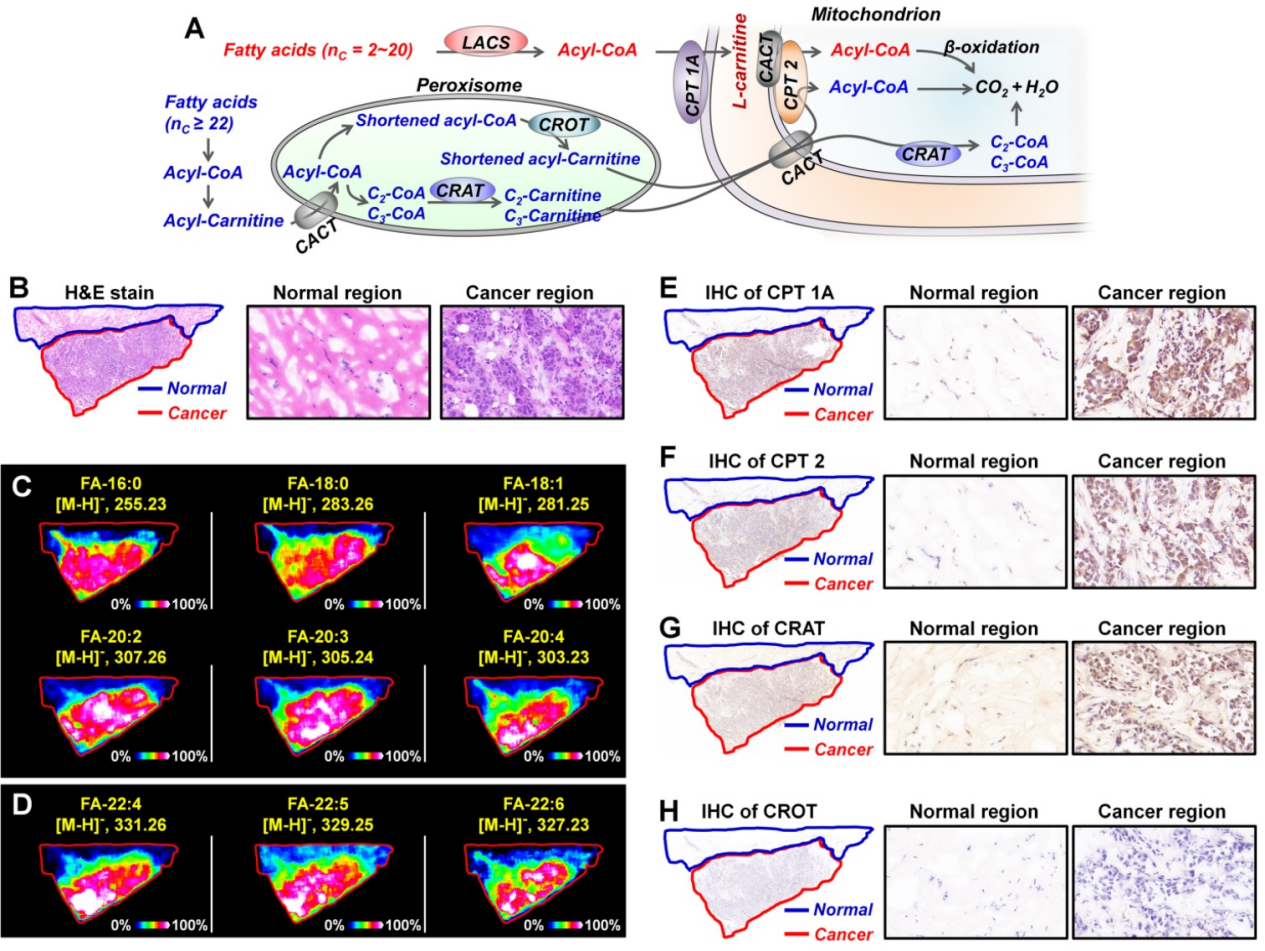
**A)** Metabolism of fatty acids in the mitochondrion and peroxisome; **B)** The H&E stain of breast cancer tissue section; **C, D)** MS images of representative fatty acids (C) and very long chain fatty acids (D) in breast cancer tissue section; **E-H)** The expressions of CPT 1A (E), CPT 2 (F), CRAT (G), and CROT (H) in breast cancer tissue section adjacent to MS imaging section. These tissue sections are from patient *No.* 535437. CACT, carnitine/acylcarnitine translocase; CPT 1A, carnitine palmitoyltransferase 1A; CPT 2, carnitine palmitoyltransferase 2; CRAT, carnitine acetyltransferase; CROT, carnitine octanoyltransferase; LCAS, long-chain acyl-CoA synthetase.

Metabolic enzymes, as nodes of biological metabolic network, can regulate and control the flux of metabolites. There is growing evidence that targeting the tumor-associated metabolic enzymes could be an effective approach to inhibit the unlimited proliferation of tumor cells 4,6. The analysis above shows that carnitine molecules are significantly altered in breast cancer. L-carnitine and acylcarnitines play obligatory roles in the β-oxidation of fatty acids, the altered carnitines may affect fatty acid metabolism 11,12. As shown in Figure 7A, for fatty acids with 2-20 carbon atoms, they are first activated by the long-chain acyl-CoA synthetase (LCAS) to form acyl-CoA, and then are translocated into the mitochondrial matrix precede their β-oxidation chain shortening with the help of carnitine dependent transport system. This transport system consists of L-carnitine and three key metabolic enzymes: carnitine palmitoyltransferase 1 (CPT 1), carnitine/acylcarnitine translocase (CACT), and carnitine palmitoyltransferase 2 (CPT 2) 46,47. However, because very long chain fatty acids (VLCFA, ≥ C_22_) cannot be transported by CPT1, they must be converted into shortened acyl-CoA in the peroxisome before they can be further transported into mitochondria (Figure 7A) 48. In the peroxisome, shortened acyl-CoA is converted to shortened acylcarnitine via the catalytic action of carnitine octanoyltransferase (CROT); C_2_-, C_3_-CoA were converted to C_2_-, C_3_-carnitine by the catalytic action of carnitine acetyltransferase (CRAT). The acylcarnitines are then transferred to the mitochondrial matrix for the following β-oxidation.

In order to investigate the expressions of fatty acids in breast cancer tissues, we carried out MALDI-MSI experiments on adjacent tissue sections in negative ion mode. Remarkable, all fatty acids, including C_16_-C_20_ fatty acids and C_22_ VLCFA, were found to be significantly upregulated in cancer tissue than the paracancerous normal tissues (Figure 7C and Figure 7D). These data suggest that carnitines and their pathway-related fatty acid oxidation metabolism may be related to the development of breast cancer. In terms of metabolic pathways, metabolites are direct substrates or products of metabolic enzymes, and their expressions in tissue can reflect enzyme capacities 49. Based on fatty acid β-oxidation pathway, CPT1, CPT 2, CRAT, and CROT are four essential enzymes for the oxidation of fatty acids 47. We speculated that the alterations of carnitines and fatty acids in breast cancer may be attributed to the abnormal expressions of these four metabolic enzymes. Therefore, we further performed targeted IHC testing of the four suspected metabolic enzymes on successive breast cancer tissue sections (adjacent to the tissue section analyzed by MALDI-MSI) to validate our discovery. Figure 7E-7H present the IHC stain images of CPT 1A, CPT 2, CRAT, and CROT in breast cancer and normal tissues. The expressions of CPT 1A, CPT 2, and CRAT in cancer tissues were found to be much higher than those in paired normal tissues, in good agreement with the upregulated carnitines and fatty acids. In fact, as the first rate-limiting enzyme in the transport of fatty acid for oxidation, CPT 1 has been attracting the attention of oncologists 50. A previous study demonstrated that CPT 1A is upregulated in human MCF-7 breast cancer cells, and targeting CPT 1A can induce apoptosis of cancer cells 51. Remarkably, CPT 2 and CRAT were found for the first time to be altered in breast cancer, and this provides new potential metabolic vulnerabilities that might be targeted for cancer therapy.

## Conclusions

In summary, we developed a sensitive and wide coverage MALDI-MSI method to visualize the spatially resolved reprogramming of carnitine metabolism in breast cancer. A total of 17 carnitines, including L-carnitine, 6 short-chain acylcarnitines, 3 middle-chain acylcarnitines, and 7 long-chain acylcarnitines can be imaged in an untargeted analysis. Metabolomics study based on the newly developed MALDI-MSI method indicates that L-carnitine and short-chain acylcarnitines are significantly altered in both human breast cancer and xenograft mouse model. A classification model built from the carnitine profiles of 170 cancer samples and 128 normal samples enabled an accurate identification of breast cancer, with overall agreement of 96.1% and 94.7% for cancer and normal tissues, respectively. Moreover, the MALDI-MSI data suggest that the β-oxidation metabolic pathway that is mediated by the carnitine system was also reprogrammed in breast cancer. Three abnormally expressed metabolic enzymes (CPT 1A, CPT 2, and CRAT) were discovered. Remarkably, CPT 2 and CRAT were found for the first time to be differentially expressed in breast cancer tissue. Thus, the spatially resolved profiling of the altered carnitine metabolism in cancer tissues will greatly expand our understanding of tumor metabolic reprogramming and may provide new potential metabolic targets for cancer therapy.

## Supplementary Material

Supplementary figures and tables.

## References

[1] Bray F, Ferlay J, Soerjomataram I, Siegel RL, Torre LA, Jemal A (2018). Global cancer statistics 2018: GLOBOCAN estimates of incidence and mortality worldwide for 36 cancers in 185 countries. CA Cancer J Clin.

[2] Hanahan D, Weinberg RA (2011). Hallmarks of cancer: the next generation. Cell.

[3] Ward PS, Thompson CB (2012). Metabolic reprogramming: a cancer hallmark even warburg did not anticipate. Cancer Cell.

[4] Vander Heiden MG, DeBerardinis RJ (2017). Understanding the Intersections between Metabolism and Cancer Biology. Cell.

[5] Martinez-Outschoorn UE, Peiris-Pages M, Pestell RG, Sotgia F, Lisanti MP (2017). Cancer metabolism: a therapeutic perspective. Nat Rev Clin Oncol.

[6] Luengo A, Gui DY, Vander Heiden MG (2017). Targeting Metabolism for Cancer Therapy. Cell Chem Biol.

[7] Hay N (2016). Reprogramming glucose metabolism in cancer: can it be exploited for cancer therapy?. Nature Reviews Cancer.

[8] Melone MAB, Valentino A, Margarucci S, Galderisi U, Giordano A, Peluso G (2018). The carnitine system and cancer metabolic plasticity. Cell Death Dis.

[9] Valentino A, Calarco A, Di Salle A, Finicelli M, Crispi S, Calogero RA (2017). Deregulation of MicroRNAs mediated control of carnitine cycle in prostate cancer: molecular basis and pathophysiological consequences. Oncogene.

[10] Claus SP (2014). Mammalian-Microbial Cometabolism of L-Carnitine in the Context of Atherosclerosis. Cell Metabolism.

[11] Yu D, Zhou L, Xuan Q, Wang L, Zhao X, Lu X (2018). Strategy for Comprehensive Identification of Acylcarnitines Based on Liquid Chromatography-High-Resolution Mass Spectrometry. Anal Chem.

[12] Jones JG, Sherry AD, Jeffrey FMH, Storey CJ, Malloy CR (1993). Sources of acetyl-CoA entering the tricarboxylic acid cycle as determined by analysis of succinate 13C isotopomers. Biochemistry.

[13] Ma K, Hutchins A, Sung S-JS, Adams MWW Pyruvate ferredoxin oxidoreductase from the hyperthermophilic archaeon, Pyrococcus furiosus, functions as a CoA-dependent pyruvate decarboxylase. Proc Natl Acad Sci U S A. 94: 9608-13.

[14] Li S, Gao D, Jiang Y (2019). Function, Detection and Alteration of Acylcarnitine Metabolism in Hepatocellular Carcinoma. Metabolites.

[15] Wang Y, Chen Y, Guan L, Zhang H, Huang Y, Johnson CH (2018). Carnitine palmitoyltransferase 1C regulates cancer cell senescence through mitochondria-associated metabolic reprograming. Cell Death Differ.

[16] Lu Y, Li N, Gao L, Xu YJ, Huang C, Yu K (2016). Acetylcarnitine Is a Candidate Diagnostic and Prognostic Biomarker of Hepatocellular Carcinoma. Cancer Res.

[17] Giesbertz P, Ecker J, Haag A, Spanier B, Daniel H An LC-MS/MS method to quantify acylcarnitine species including isomeric and odd-numbered forms in plasma and tissues. J Lipid Res. 56: 2029-39.

[18] Ganti S, Taylor SL, Kim K, Hoppel CL, Guo L, Yang J (2012). Urinary acylcarnitines are altered in human kidney cancer. Int J Cancer.

[19] Caprioli RM, Farmer TB, Gile J (1997). Molecular imaging of biological samples: localization of peptides and proteins using MALDI-TOF MS. Anal Chem.

[20] Caprioli RM (2016). Imaging mass spectrometry: Molecular microscopy for the new age of biology and medicine. Proteomics.

[21] Aichler M, Walch A (2015). MALDI Imaging mass spectrometry: current frontiers and perspectives in pathology research and practice. Lab Invest.

[22] Addie RD, Balluff B, Bovee JV, Morreau H, McDonnell LA (2015). Current State and Future Challenges of Mass Spectrometry Imaging for Clinical Research. Anal Chem.

[23] Tang W, Chen J, Zhou J, Ge J, Zhang Y, Li P (2019). Quantitative MALDI Imaging of Spatial Distributions and Dynamic Changes of Tetrandrine in Multiple Organs of Rats. Theranostics.

[24] Lin Z, Cai Z (2018). Negative ion laser desorption/ionization time-of-flight mass spectrometric analysis of small molecules by using nanostructured substrate as matrices. Mass Spectrom Rev.

[25] Kompauer M, Heiles S, Spengler B (2017). Atmospheric pressure MALDI mass spectrometry imaging of tissues and cells at 1.4-mum lateral resolution. Nat Methods.

[26] Baker TC, Han J, Borchers CH (2017). Recent advancements in matrix-assisted laser desorption/ionization mass spectrometry imaging. Curr Opin Biotechnol.

[27] Mirnezami R, Spagou K, Vorkas PA, Lewis MR, Kinross J, Want E (2014). Chemical mapping of the colorectal cancer microenvironment via MALDI imaging mass spectrometry (MALDI-MSI) reveals novel cancer-associated field effects. Mol Oncol.

[28] Calligaris D, Feldman DR, Norton I, Olubiyi O, Changelian AN, Machaidze R (2015). MALDI mass spectrometry imaging analysis of pituitary adenomas for near-real-time tumor delineation. Proc Natl Acad Sci U S A.

[29] Hall Z, Ament Z, Wilson CH, Burkhart DL, Ashmore T, Koulman A (2016). Myc Expression Drives Aberrant Lipid Metabolism in Lung Cancer. Cancer Res.

[30] Mascini NE, Eijkel GB, ter Brugge P, Jonkers J, Wesseling J, Heeren RMA (2015). The Use of Mass Spectrometry Imaging to Predict Treatment Response of Patient-Derived Xenograft Models of Triple-Negative Breast Cancer. J Proteome Res.

[31] Wang J, Qiu S, Chen S, Xiong C, Liu H, Wang J (2015). MALDI-TOF MS Imaging of Metabolites with a N-(1-Naphthyl) Ethylenediamine Dihydrochloride Matrix and Its Application to Colorectal Cancer Liver Metastasis. Anal Chem.

[32] McDonnell LA, Angel PM, Lou S, Drake RR (2017). Chapter Eleven - Mass Spectrometry Imaging in Cancer Research: Future Perspectives. In: Drake RR, McDonnell LA, editors. Adv Cancer Res: Academic Press.

[33] Randall EC, Lopez BGC, Peng S, Regan MS, Abdelmoula WM, Basu SS (2019). Localized Metabolomic Gradients in Patient-Derived Xenograft Models of Glioblastoma. Cancer Res.

[34] Chughtai K, Jiang L, Greenwood TR, Glunde K, Heeren RMA (2012). Mass Spectrometry Images Acylcarnitines, Phosphatidylcholines and Sphingomyelin in MDA-MB-231 Breast Tumor Models. J Lipid Res.

[35] Li T, He J, Mao X, Bi Y, Luo Z, Guo C (2015). In situ biomarker discovery and label-free molecular histopathological diagnosis of lung cancer by ambient mass spectrometry imaging. Sci Rep.

[36] Sun C, Liu W, Mu Y, Wang X (2020). 1,1′-binaphthyl-2,2′-diamine as a novel MALDI matrix to enhance the in situ imaging of metabolic heterogeneity in lung cancer. Talanta.

[37] Yano H, Oyanagi E, Kato Y, Samejima Y, Sasaki J, Utsumi K (2010). L-carnitine is essential to beta-oxidation of quarried fatty acid from mitochondrial membrane by PLA(2). Mol Cell Biochem.

[38] Lou S, Balluff B, Cleven AHG, Bovée JVMG, McDonnell LA (2017). Prognostic Metabolite Biomarkers for Soft Tissue Sarcomas Discovered by Mass Spectrometry Imaging. J Am Soc Mass Spectrom.

[39] Seeley EH, Oppenheimer SR, Mi D, Chaurand P, Caprioli RM (2008). Enhancement of protein sensitivity for MALDI imaging mass spectrometry after chemical treatment of tissue sections. J Am Soc Mass Spectrom.

[40] Sun C, Li Z, Ma C, Zang Q, Li J, Liu W (2019). Acetone immersion enhanced MALDI-MS imaging of small molecule metabolites in biological tissues. J Pharm Biomed Anal.

[41] Angel PM, Spraggins JM, Baldwin HS, Caprioli R (2012). Enhanced Sensitivity for High Spatial Resolution Lipid Analysis by Negative Ion Mode Matrix Assisted Laser Desorption Ionization Imaging Mass Spectrometry. Anal Chem.

[42] Tucker LH, Hamm GR, Sargeant RJE, Goodwin RJA, Mackay CL, Campbell CJ (2019). Untargeted Metabolite Mapping in 3D Cell Culture Models Using High Spectral Resolution FT-ICR Mass Spectrometry Imaging. Anal Chem.

[43] Enot DP, Lin W, Beckmann M, Parker D, Overy DP, Draper J (2008). Preprocessing, classification modeling and feature selection using flow injection electrospray mass spectrometry metabolite fingerprint data. Nat Protoc.

[44] Sun C, Li T, Song X, Huang L, Zang Q, Xu J (2019). Spatially resolved metabolomics to discover tumor-associated metabolic alterations. Proc Natl Acad Sci U S A.

[45] Xu J, Chen Y, Zhang R, Song Y, Cao J, Bi N (2013). Global and targeted metabolomics of esophageal squamous cell carcinoma discovers potential diagnostic and therapeutic biomarkers. Mol Cell Proteomics.

[46] Hoppel C Fatty acid import into mitochondria Biochim Biophys Acta 2000 1486017 10.1016/s1388-1981(00)00044-5 10856709

[47] Wanders RJA (1999). Molecular cloning and expression of human carnitine octanoyltransferase: evidence for its role in the peroxisomal beta-oxidation of branched-chain fatty acids. Biochem Biophys Res Commun.

[48] Bieber LL (1993). Properties of the medium chain/Long chain carnitine acyltransferase purified from rat liver microsomes. J Biol Chem.

[49] Fendt SM, Buescher JM, Rudroff F, Picotti P, Zamboni N, Sauer U (2010). Tradeoff between enzyme and metabolite efficiency maintains metabolic homeostasis upon perturbations in enzyme capacity. Mol Syst Biol.

[50] Currie E, Schulze A, Zechner R, Walther TC, Farese RV Jr (2013). Cellular fatty acid metabolism and cancer. Cell Metab.

[51] Pucci S, Zonetti MJ, Fisco T, Polidoro C, Bocchinfuso G, Palleschi A (2016). Carnitine palmitoyl transferase-1A (CPT1A): a new tumor specific target in human breast cancer. Oncotarget.

