# Supplementary material for: Mass spectrometry imaging-based metabolomics to visualize the spatially resolved reprogramming of carnitine metabolism in breast cancer
Source: Theranostics. 2020 May 30;10(16):7070–82. doi: 10.7150/thno.45543 (PMC7330837; doi:10.7150/thno.45543)
Supplement: Supplementary file 1 — Supplementary figures and tables. [file thnov10p7070s1.pdf]

Supplementary data

Mass spectrometry imaging-based metabolomics to visualize the spatially resolved reprogramming of carnitine metabolism in breast cancer

Chenglong Sun[1,2*], Fukai Wang[3], Yang Zhang[4], Jinqian Yu[1,2], Xiao Wang[1,2*]

1. School of Pharmaceutical Sciences, Qilu University of Technology (Shandong Academy of Sciences), Jinan 250014, China.
2. Shandong Analysis and Test Center, Qilu University of Technology (Shandong Academy of Sciences), Jinan 250014, China.
3. Shandong Cancer Hospital and Institute, Shandong First Medical University and Shandong Academy of Medical Sciences, Jinan 250117, China.
4. Department of Ultrasound in Medicine, Shanghai Jiao Tong University Affiliated Sixth People's Hospital, Shanghai 200233, China.

* Corresponding authors: E-mail: wangx@sdas.org; chenglongsun1989@163.com

Analyte identification

The MS/MS experiment was carried out by performing LC-MS/MS analysis on Q-Qrbitrap mass spectrometer (Q Exactive, Thermo Scientific Bremen, Germany). Breast cancer tissue was weighed at approximately 50 mg and homogenated in 0.5 mL normal saline. Then 1000 μL acetonitrile was added to 200 μL homogenate and vortexed for 3 minutes. After centrifugation at 10000 rpm for 5 min, the supernatant was transfered to a sample vial for LC-MS/MS analysis. The ions of interest were listed as the targets, with the NCE value set at 25%, 35%, and 45% in targeted-MS2 scan mode. The resolving power was set at 17500 for MS/MS acquisition with the AGC value at 3E6 and maximum injection time at 200 ms. Representative results were shown in Figure S14 ~ Figure S23.

Segmentation analysis

The segmentation procedure including: (*i*) construct MS image of a certain tissue section; (*ii*) running the Tool "Find Peaks", select work on all individual spectra; (*iii*) moving the results by running "Move Peaks to Local Max"; (*iv*) using the modified result to perform segmentation with bisecting k-means, weak denoising, and Correlation Distance; (*v*) visualizing the Segmentation Map in "Labels" window. We have added this procedure in the revised manuscript.

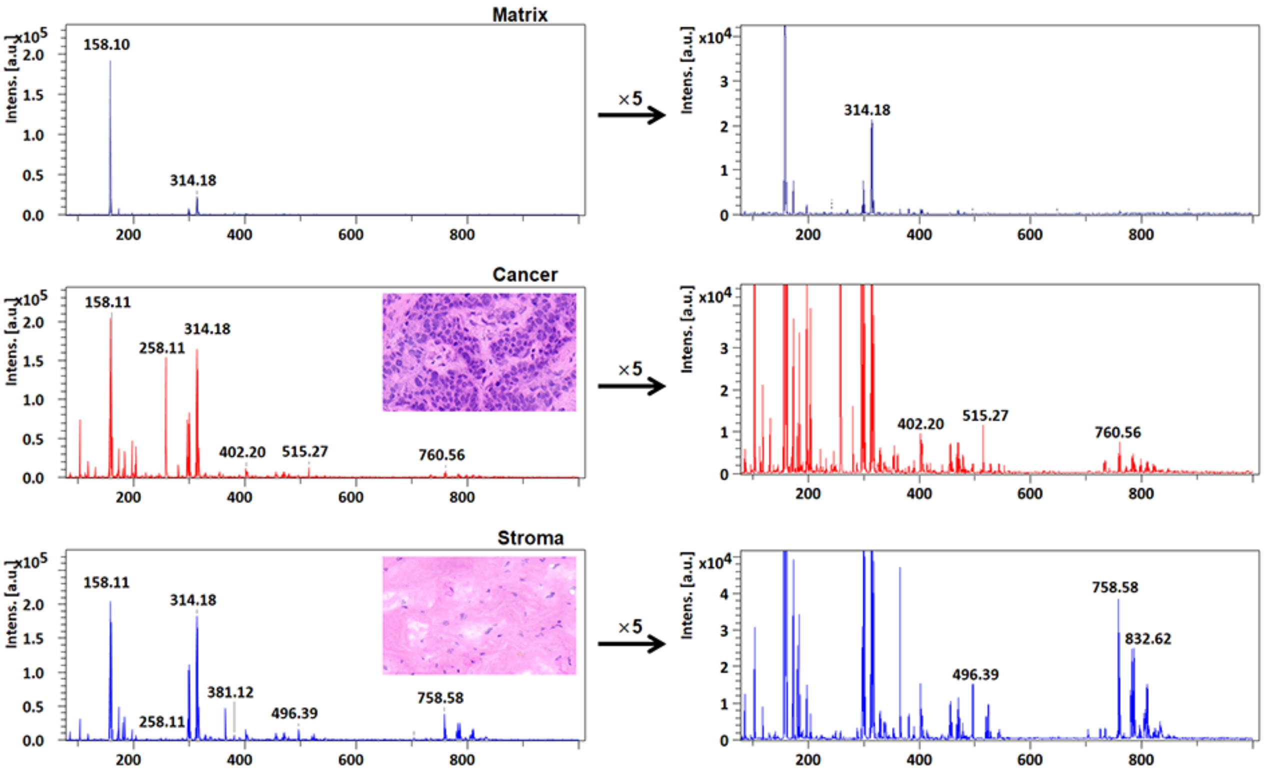

Figure S1. MALDI-MS spectra of matrix, cancer tissue, and normal tissue in breast cancer tissue section.

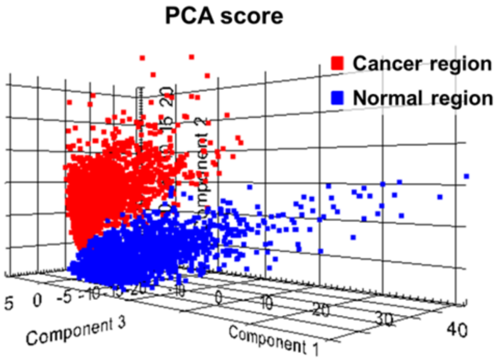

Figure S2. The PCA score plot based on the *in situ* MALDI-MS spectra of breast cancer regions and adjacent normal regions.

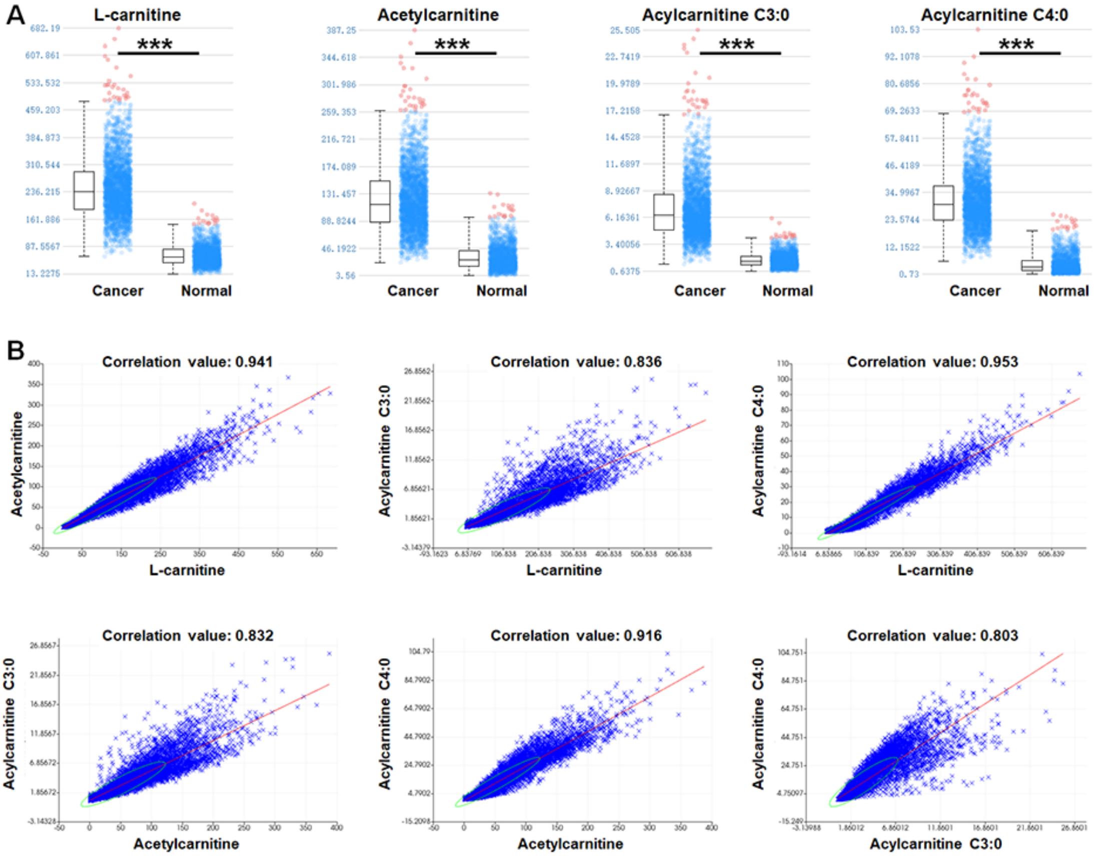

Figure S3. **A)** Calculated intensities of four carnitines in breast cancer regions and adjacent normal region; **B)** Correlation analysis of four carnitines in all pixels of the breast cancer tissue section. (The statistical data comes from all pixels of sample *No*. 535905); ***, $p < 0.001$.

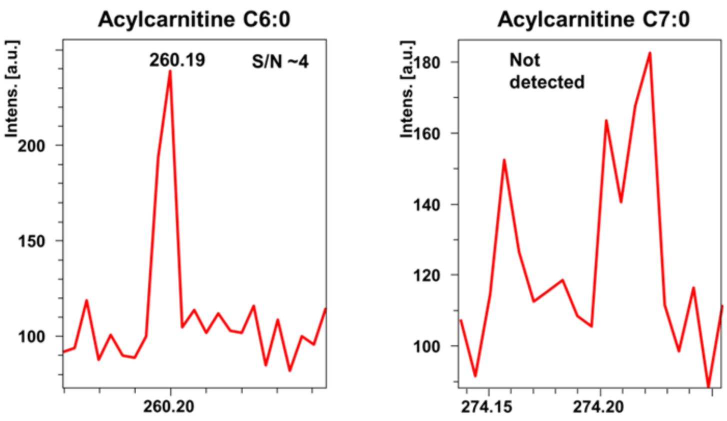

Figure S4. MS spectra of acylcarnitine C6:0 and acylcarnitine C7:0 in breast cancer tissue section.

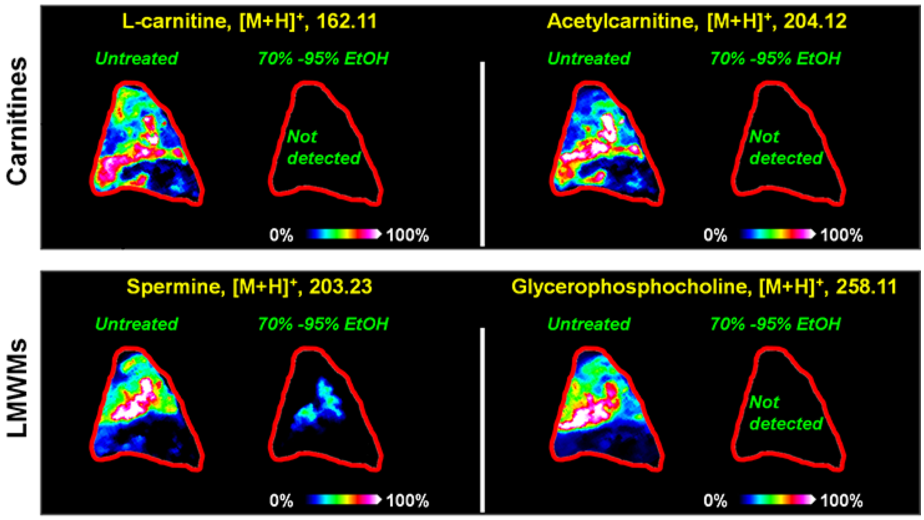

Figure S5. MS images of representative carnitines and other low-molecular-weight metabolites (LMWMs) in untreated and 70% - 95% ethanol-washed breast cancer tissue sections.

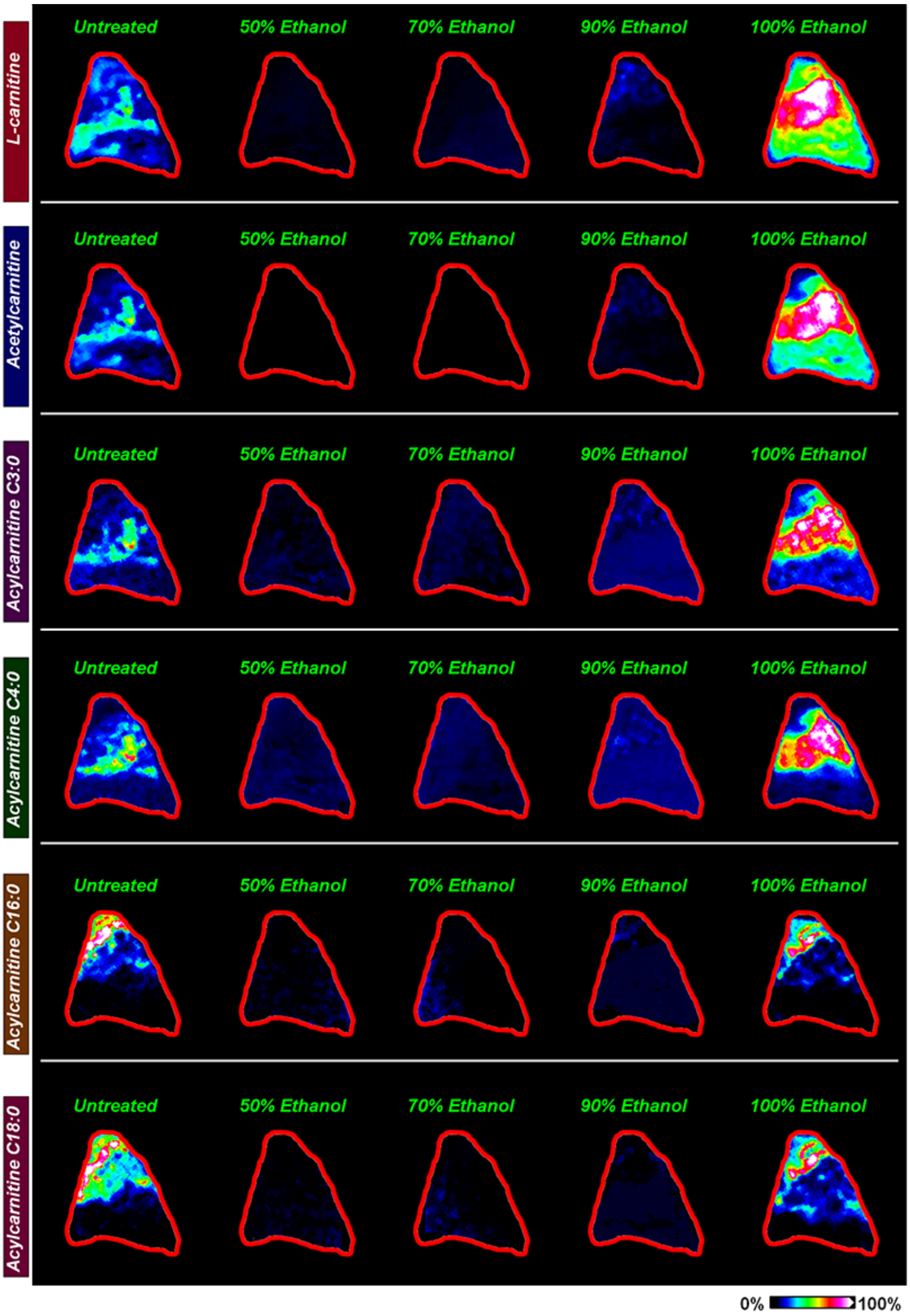

Figure S6. MS images of representative carnitines in untreated, 50% ethanol-, 70% ethanol-, 90% ethanol-, and 100% ethanol-washed breast cancer tissue sections.

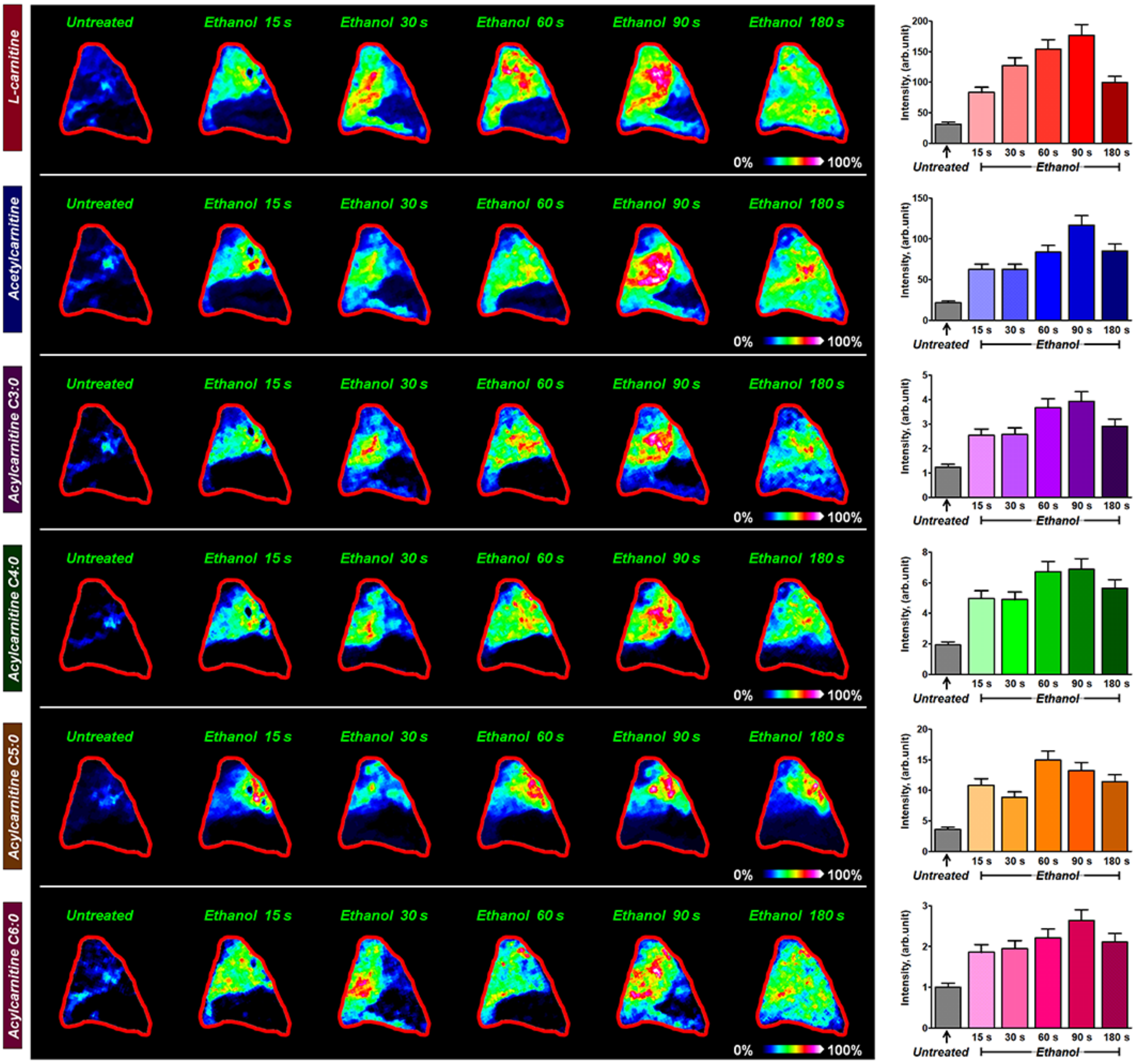

Figure S7. MS images and statistical data of representative carnitines in untreated and 100% ethanol-washed breast cancer tissue sections.

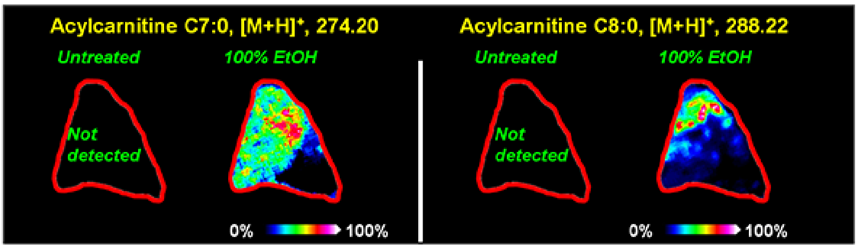

Figure S8. MS images of acylcarnitine C7:0 and acylcarnitine C8:0 in untreated and 100% ethanol-washed breast cancer tissue sections.

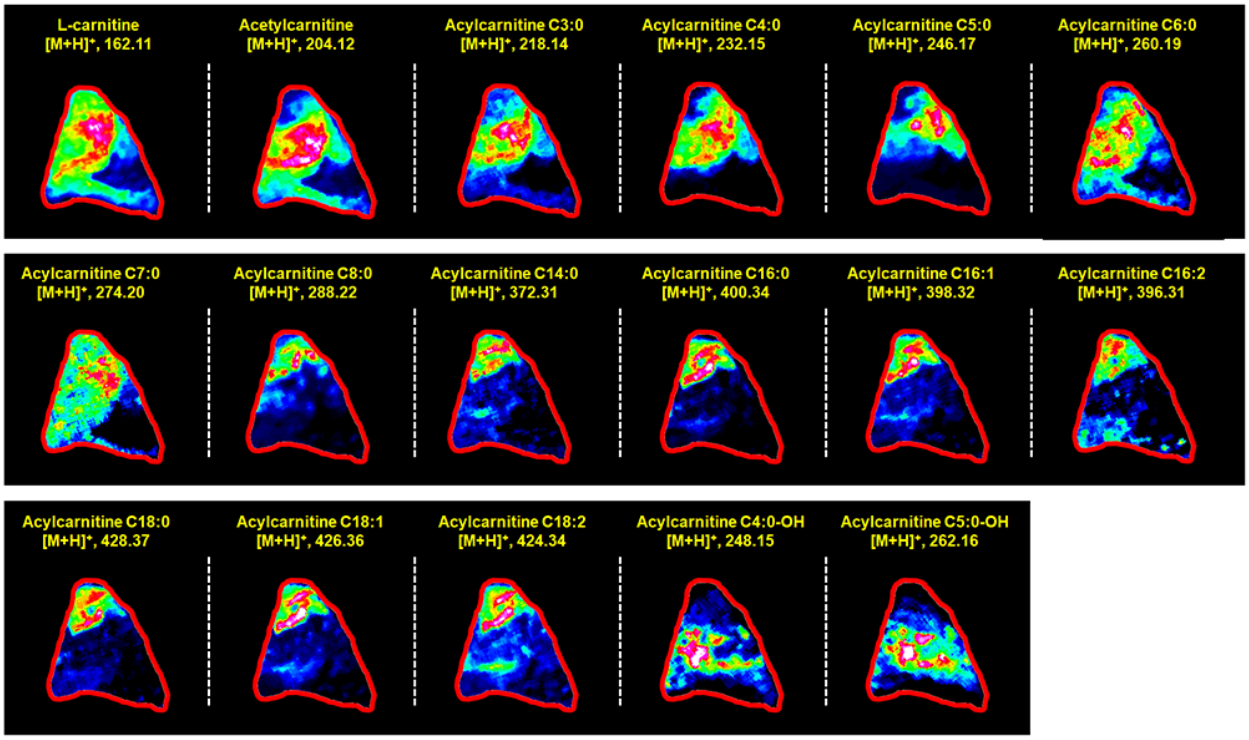

Figure S9. MS images of 17 detected carnitines in 100% ethanol-washed breast cancer tissue sections.

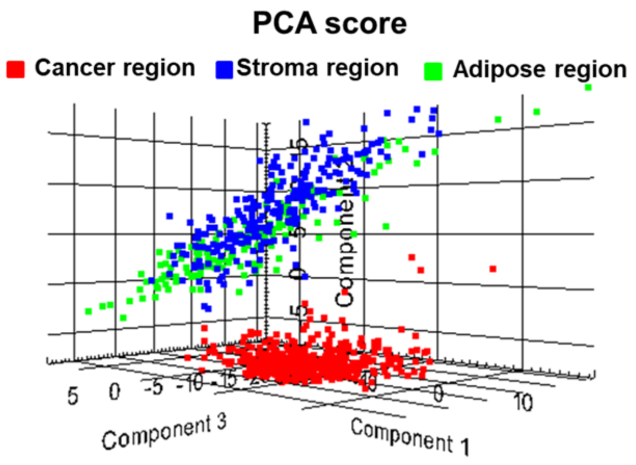

Figure S10. The PCA score plots based on the *in situ* MALDI-MS spectra of cancer regions, stroma regions, and adipose regions (The statistical data comes from all pixels of mouse breast cancer tissues *No*. R001).

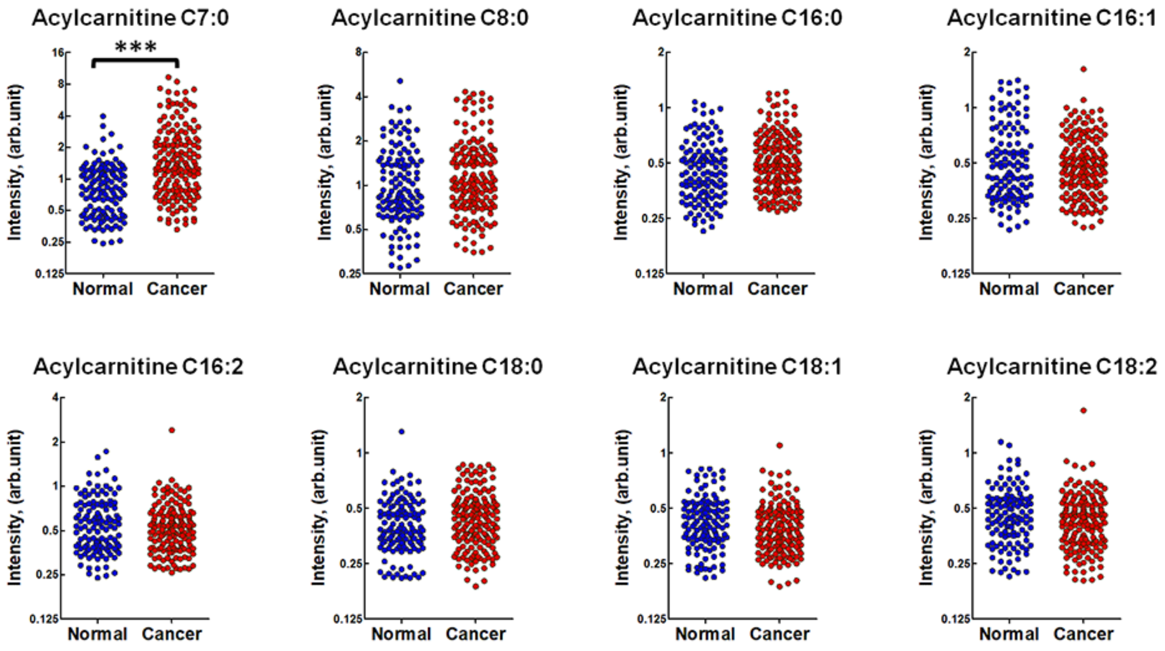

Figure S11. Statistical data of eight representative acylcarnitines in human breast cancer tissues. The scale of y-axis is log 2; ***, $p < 0.001$.

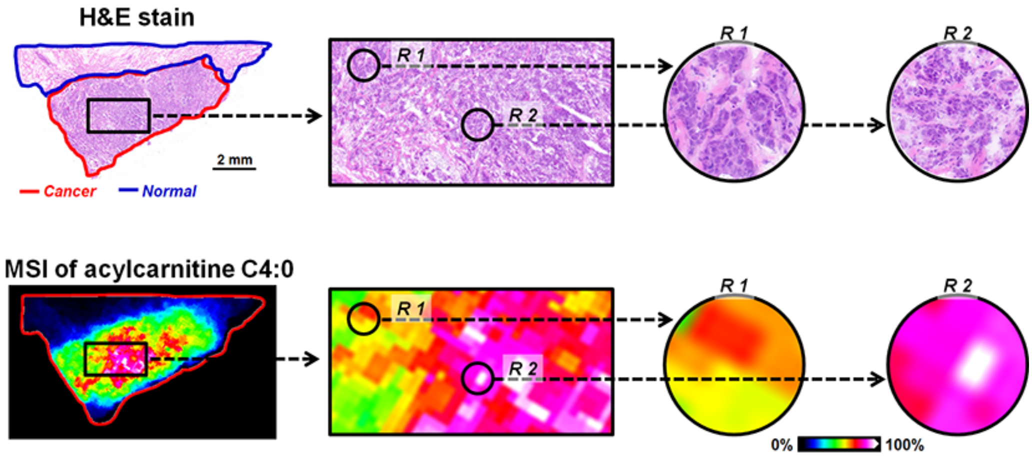

Figure S12. The content of acylcarnitine C4:0 are not completely consistent in the same cancer region.

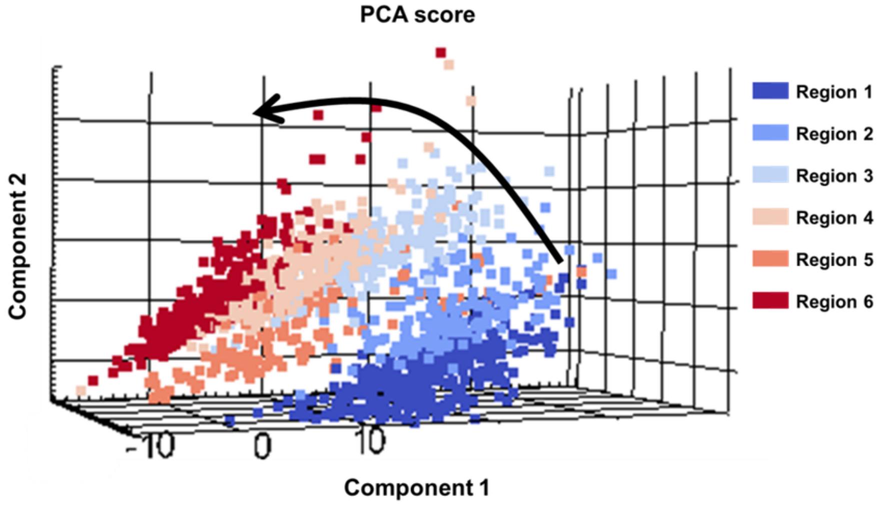

Figure S13. The PCA score plot based on the metabolite profiles of six segmentation-derived regions. The statistical data comes from all pixels of sample *No.* 535437.

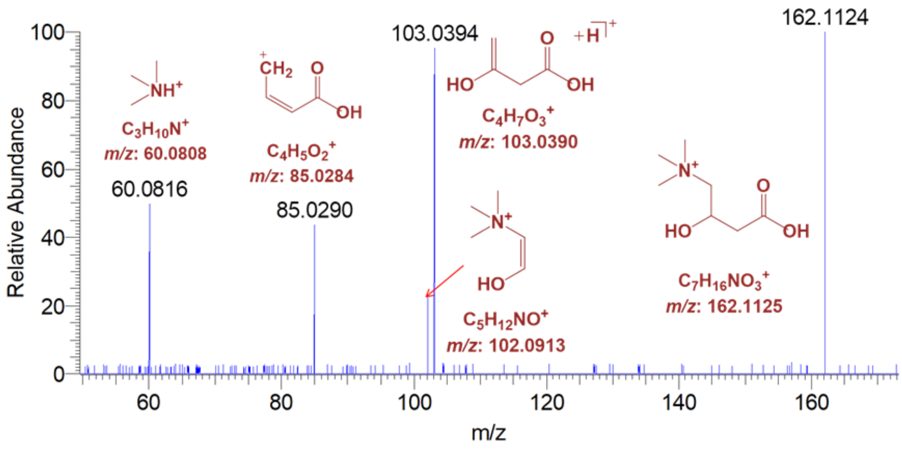

Figure S14. The MS/MS spectrum of L-carnitine.

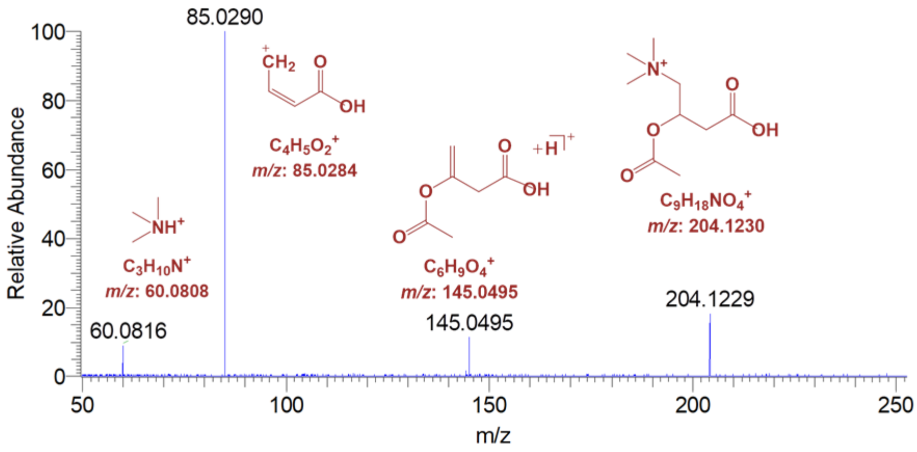

Figure S15. The MS/MS spectrum of acetylcarnitine.

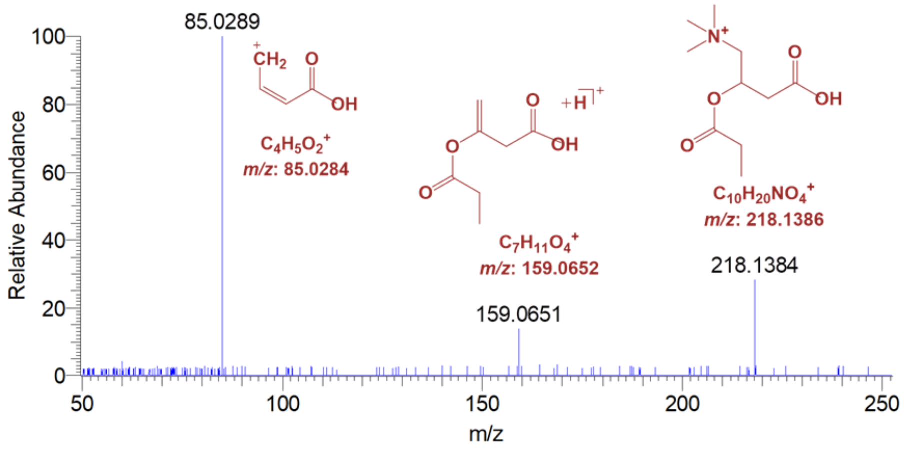

Figure S16. The MS/MS spectrum of acylcarnitine C3:0.

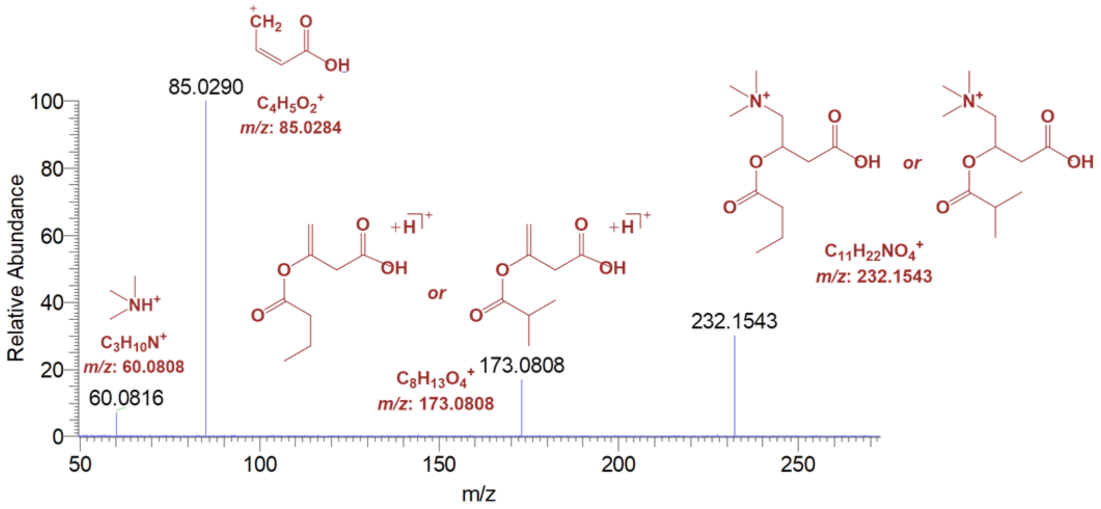

Figure S17. The MS/MS spectrum of acylcarnitine C4:0.

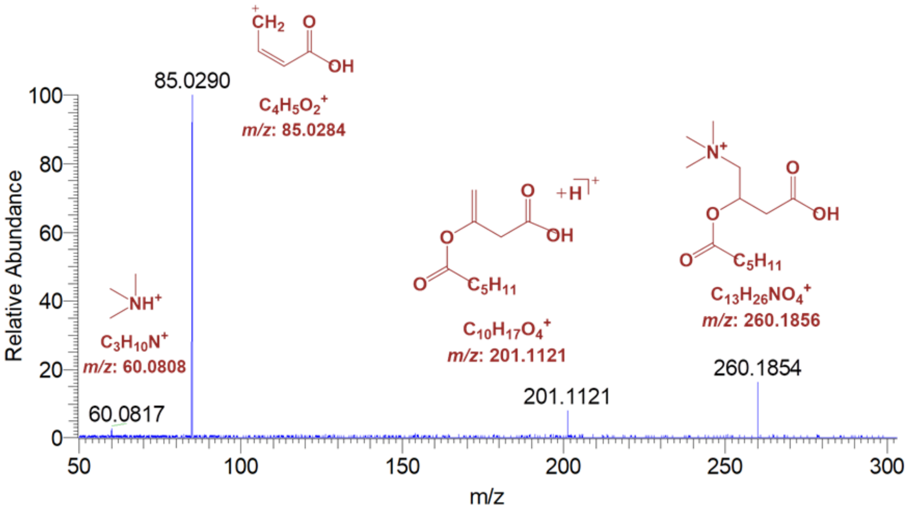

Figure S18. The MS/MS spectrum of acylcarnitine C6:0.

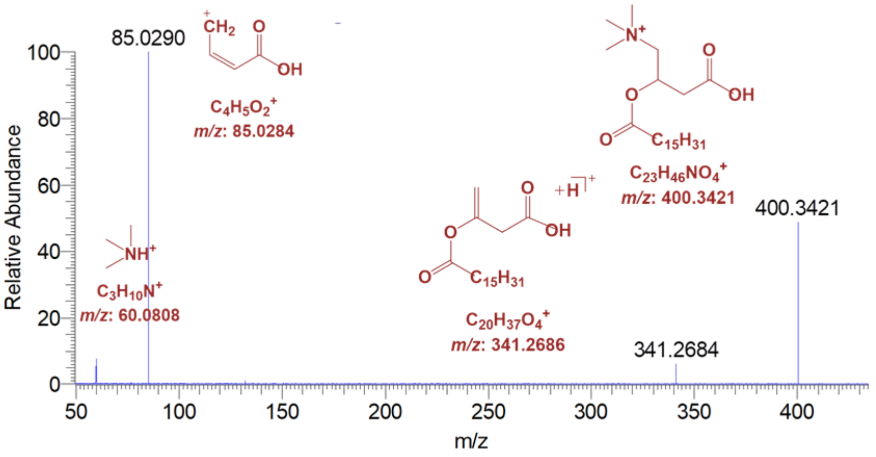

Figure S19. The MS/MS spectrum of acylcarnitine C16:0.

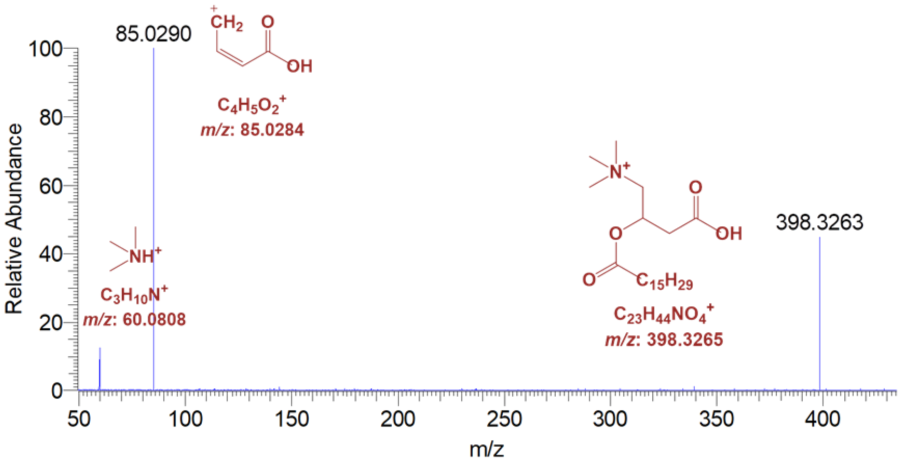

Figure S20. The MS/MS spectrum of acylcarnitine C16:1.

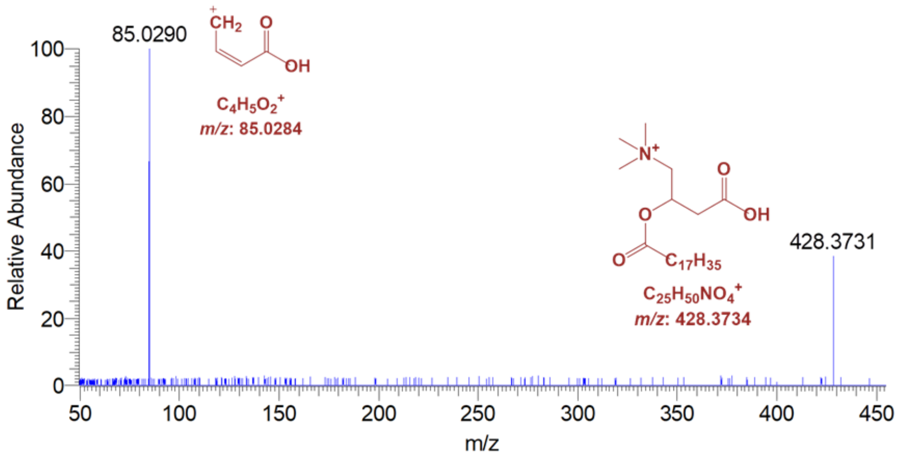

Figure S21. The MS/MS spectrum of acylcarnitine C18:0.

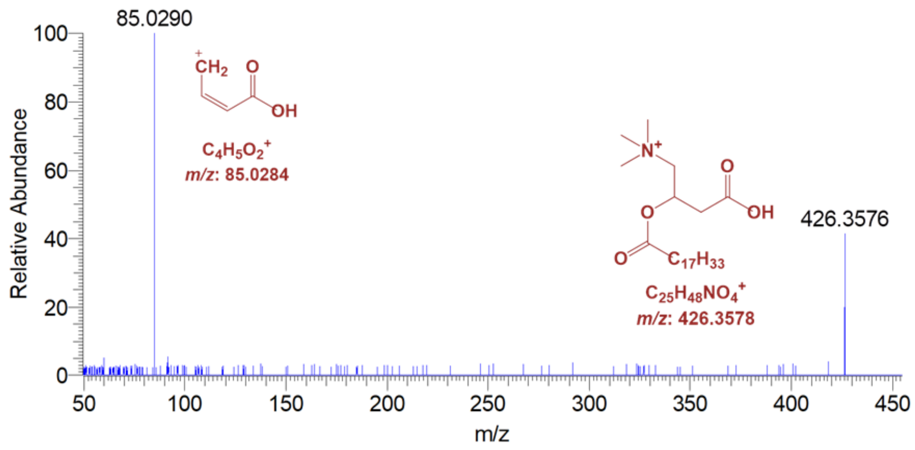

Figure S22. The MS/MS spectrum of acylcarnitine C18:1.

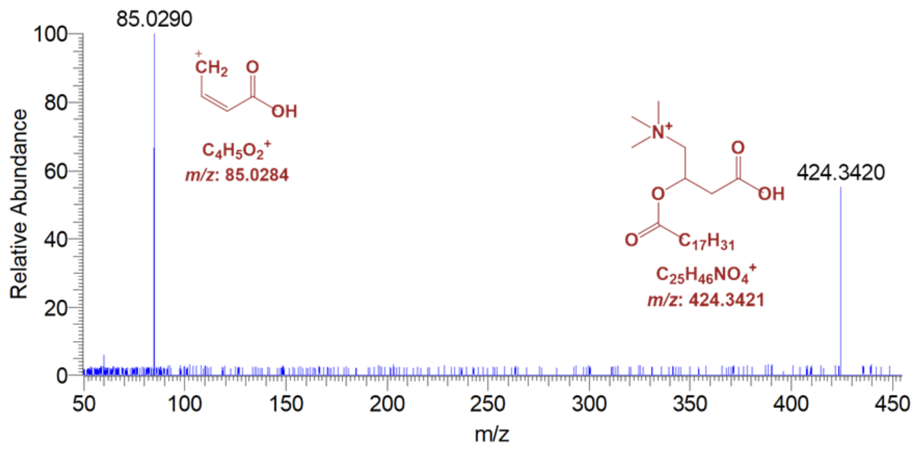

Figure S23. The MS/MS spectrum of acylcarnitine C18:2.

Table S1. Demographic and characteristics of 58 breast cancer patients.

No.	Age (year)	Lesion	Tumor grade	Metastasis	Previous treatment
535431	56	Left breast	T3	No Metastasis	No
535437	44	Left breast	T2	No Metastasis	No
535425	53	Left breast	T2	No Metastasis	No
535466	42	Left breast	T3	No Metastasis	No
535512	49	Left breast	T2	No Metastasis	No
535673	69	Right breast	T3	No Metastasis	No
535905	40	Right breast	T3	No Metastasis	No
535930	53	Right breast	T2	No Metastasis	No
536171	48	Right breast	T3	No Metastasis	No
536190	44	Left breast	T1	No Metastasis	No
536208	51	Left breast	T2	No Metastasis	No
536286	48	Left breast	T2	No Metastasis	No
536348	47	Left breast	T3	No Metastasis	No
536360	39	Right breast	T3	No Metastasis	Preoperative chemotherapy
536262	46	Left breast	T2	No Metastasis	No
536503	63	Left breast	T3	Metastasis	No
536522	65	Right breast	T3	No Metastasis	No
536613	58	Right breast	T1	No Metastasis	No
536645	66	Left breast	T2	No Metastasis	No
536704	49	Left breast	T2	No Metastasis	No
536719	59	Left breast	T2	No Metastasis	No
536746	65	Left breast	T3	No Metastasis	No
536916	69	Right breast	T3	No Metastasis	No
537144	43	Right breast	T2	No Metastasis	No
537187	49	Right breast	T3	Metastasis	No
537234	62	Left breast	T1	No Metastasis	No
537266	59	Right breast	T3	No Metastasis	No
537309	68	Left breast	T2	No Metastasis	No
537355	62	Left breast	T2	No Metastasis	No
537411	59	Right breast	T1	No Metastasis	No
537435	67	Right breast	T3	No Metastasis	Preoperative chemotherapy
537524	70	Right breast	T2	No Metastasis	No
537533	59	Left breast	T2	No Metastasis	No
537603	65	Left breast	T2	No Metastasis	No
537629	61	Left breast	T3	No Metastasis	No
537671	69	Right breast	T2	No Metastasis	No
537739	40	Right breast	T3	No Metastasis	No
537754	57	Left breast	T1	No Metastasis	No
537807	49	Right breast	T3	No Metastasis	No
537829	67	Left breast	T3	No Metastasis	Preoperative chemotherapy
537857	59	Left breast	T2	No Metastasis	No
537902	71	Left breast	T2	No Metastasis	No
537933	64	Right breast	T3	No Metastasis	No
537965	48	Left breast	T2	No Metastasis	No
538008	64	Left breast	T2	No Metastasis	No
538025	62	Right breast	T3	Metastasis	No
538054	52	Left breast	T2	No Metastasis	No
538107	64	Right breast	T2	No Metastasis	No
538144	68	Right breast	T3	No Metastasis	No
538163	63	Left breast	T2	No Metastasis	No
548305	47	Right breast	T2	No Metastasis	No
548337	53	Left breast	T3	No Metastasis	Preoperative chemotherapy

548412	69	Left breast	T2	No Metastasis	No
548476	63	Left breast	T3	No Metastasis	No
548508	56	Right breast	T1	No Metastasis	No
548559	52	Left breast	T2	No Metastasis	No
548602	68	Left breast	T3	No Metastasis	No
548635	62	Left breast	T3	No Metastasis	No
